# TRC8-dependent degradation of hepatitis C virus immature core protein regulates viral propagation and pathogenesis

**DOI:** 10.1038/ncomms11379

**Published:** 2016-05-04

**Authors:** Sayaka Aizawa, Toru Okamoto, Yukari Sugiyama, Takahisa Kouwaki, Ayano Ito, Tatsuya Suzuki, Chikako Ono, Takasuke Fukuhara, Masahiro Yamamoto, Masayasu Okochi, Nobuhiko Hiraga, Michio Imamura, Kazuaki Chayama, Ryosuke Suzuki, Ikuo Shoji, Kohji Moriishi, Kyoji Moriya, Kazuhiko Koike, Yoshiharu Matsuura

**Affiliations:** 1 Department of Molecular Virology, Osaka University, Osaka, 565-0871 Japan; 2 Department of Immunoparasitology, Research Institute for Microbial Diseases, Osaka University, Osaka 565-0871, Japan; 3 Neuropsychiatry and Neurochemistry, Department of Integrated Medicine, Osaka University, Osaka 565-0871, Japan; 4 Department of Medicine and Molecular Science, Hiroshima University School of Medicine, Hiroshima 734-8551, Japan; 5 Department of Virology II, National Institute of Infectious Diseases, Tokyo 162-8640, Japan; 6 Division of Infectious Diseases Control, Center for Infectious Diseases, Kobe University Graduate School of Medicine, Kobe 650-0017, Japan; 7 Department of Microbiology, Faculty of Medicine, University of Yamanashi, Yamanashi 409-3898, Japan; 8 Department of Gastroenterology, Graduate School of Medicine, The University of Tokyo, Tokyo 113-8655, Japan

## Abstract

Signal-peptide peptidase (SPP) is an intramembrane protease that participates in the production of the mature core protein of hepatitis C virus (HCV). Here we show that SPP inhibition reduces the production of infectious HCV particles and pathogenesis. The immature core protein produced in SPP-knockout cells or by treatment with an SPP inhibitor is quickly degraded by the ubiquitin–proteasome pathway. Oral administration of the SPP inhibitor to transgenic mice expressing HCV core protein (CoreTg) reduces the expression of core protein and ameliorates insulin resistance and liver steatosis. Moreover, the haploinsufficiency of SPP in CoreTg has similar effects. TRC8, an E3 ubiquitin ligase, is required for the degradation of the immature core protein. The expression of the HCV core protein alters endoplasmic reticulum (ER) distribution and induces ER stress in SPP/TRC8 double-knockout cells. These data suggest that HCV utilizes SPP cleavage to circumvent the induction of ER stress in host cells.

## Abstract (web summary)

A cellular protease, SPP, participates in production of the mature core protein of hepatitis C virus (HCV). Here, the authors show in mouse models that SPP inhibition reduces viral propagation and pathogenesis via proteasomal degradation of the immature core protein mediated by the E3 ubiquitin ligase TRC8.

## Graphical abstract

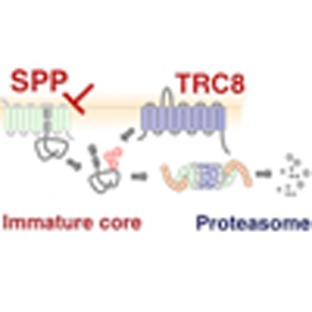

Signal-peptide peptidase (SPP) is a nine transmembrane protein that belongs to the GxGD-type intramembrane cleaving proteases^1^. SPP is required for the generation of peptide ligands for a histocompatibility antigen, α chain E (HLA-E)^2^, and the maturation of core proteins of hepatitis C virus (HCV)^3^,^4^ and equine hepacivirus (EHcV)^5^. SPP was also reported to recognize haem oxygenase-1 (HO-1)^6^,^7^ and the unspliced variant of X-box binding protein 1 (XBP1 μ)^8^ as substrates. Although it has been suggested that SPP is involved in the endoplasmic reticulum (ER)-associated degradation (ERAD) process through interaction with UBAC2 (ref. 8), PDI (ref. 9, TRC8 (ref. 10 or Derlin1 (ref. 8), the physiological functions of SPP in ERAD remain largely unknown.

HCV belongs to the Flaviviridae family and possesses a single positive-strand RNA that encodes a single polyprotein of ∼3,000 amino acids that is processed into 10 viral proteins by viral and host proteases^11^,^12^,^13^. The core protein is the first viral protein to be translated and cleaved from the precursor polyprotein by a host signal peptidase at amino-acid position 191/192 (ref. 14). The immature core protein is further processed by SPP at the C-terminal transmembrane region to form the mature core protein^3^. The maturation of the core protein by SPP is crucial for the production of infectious HCV particles^15^,^16^. Although the mature core protein participates in particle formation, transgenic mice expressing the HCV core protein in the liver (CoreTg) developed insulin resistance^17^, steatosis^18^ and hepatocellular carcinoma^19^. The levels of core protein in CoreTg livers were equivalent to those of HCV patients^19^, suggesting that the HCV core protein also plays crucial roles in HCV pathogenesis. The C terminus of the mature HCV core protein was shown to be Phe177 in insect cells^20^ and mammalian cells^15^. Mutation of the HCV core at Phe177 abolished cleavage by SPP and impaired infectious viral particle production^15^. However, the biological significance of cleavage of the HCV core protein by SPP on virus production and pathogenesis remains unknown.

In this study, we generated SPP gene-knockout (SPPKO) cell lines and mice to investigate the roles of SPP on HCV propagation and pathogenesis. We found that the immature HCV core protein produced in SPPKO cells or cells treated with an SPP inhibitor was quickly degraded by the ubiquitin–proteasome pathway. We demonstrated that the administration of an SPP inhibitor to CoreTg and single-allele deletion of SPP genes in CoreTg reduced the expression of the core protein and ameliorated insulin resistance and liver steatosis. Moreover, the production of infectious HCV was severely impaired in SPPKO cells. siRNA-mediated screening revealed that the TRC8 gene, which encodes an ER-resident E3 ubiquitin-ligase, was responsible for the degradation of the immature HCV core protein. Finally, we found that expression of the HCV core protein induced an alteration of the ER structure and ER stress in cells in which both the SPP and TRC8 genes have been knocked out (SPP/TRC8DKO). The recovery of either SPP or TRC8 expression abrogated the induction of ER stress in SPP/TRC8DKO cells, suggesting that the immature HCV core protein retained in the ER membrane induces ER stress. Taken together, our data indicate that the inhibition of SPP activity induces the production of the immature HCV core protein, and TRC8 is involved in the degradation of the immature core protein by the proteasome to circumvent the induction of ER stress.

## Results

### SPP is crucial for the expression of mature HCV core protein

**Figure 1:**
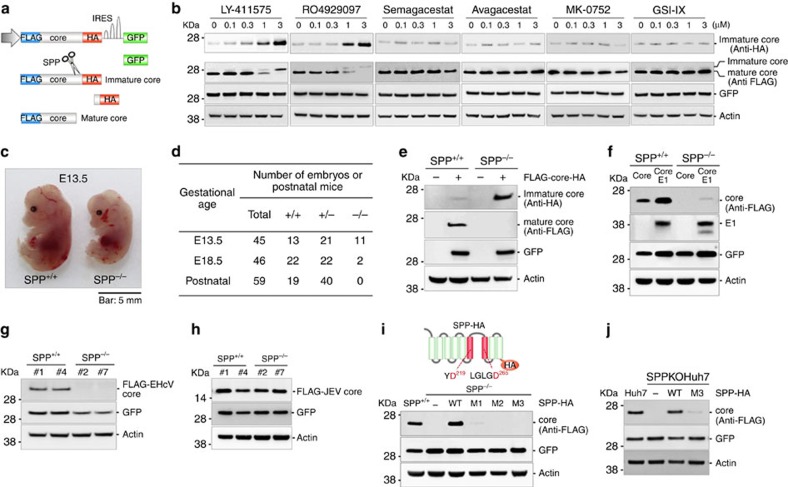
SPP activity is crucial for the stable expression of mature HCV core protein. (**a**) Structure of a cDNA clone encoding FLAG-core-HA under the ubiquitin promoter and GFP under the EMCV internal ribosomal entry site (IRES). When processing of the HCV core protein was inhibited by SPP, staining with anti-HA antibody revealed the immature core protein. (**b**) Huh7 cells expressing FLAG-core-HA were treated with various γ-secretase inhibitors at concentrations of 0.1, 0.3, 1.0 or 3 μM for 2 days. (**c**) SPP^+/+^ and SPP^−/−^ embryos at E13.5 arising from SPP heterozygous crosses. Scale bar, 5 mm. (**d**) Genotyping of embryos arising from SPP heterozygous crosses. (**e**) The expression levels of FLAG-core-HA in SPP^+/+^ and SPP^−/−^ MEFs at 48 h post transfection are shown. (**f**) The expression levels of FLAG-core or FLAG-core-E1 in SPP^+/+^ and SPP^−/−^ MEFs at 48 h post transfection are shown. (**g**) cDNAs encoding FLAG-tagged equine hepacivirus core (FLAG-EHcV-core) were introduced into SPP^+/+^ (#1 and #4) and SPP^−/−^ (#2 and #7) MEFs. (**h**) A cDNA encoding FLAG-tagged JEV core (FLAG-JEV core) was introduced into SPP^+/+^ (#1 and #4) and SPP^−/−^ (#2 and #7) MEFs. (**i**) Transmembrane regions 6 and 7 in SPP (indicated in red) encode peptidase activity to cleave substrates (upper). SPP^+/+^ and SPP^−/−^ MEFs stably expressing HA-tagged SPP wild type (WT), SPP-HA D219A (mutant 1; M1), SPP-HA D265A (mutant 2; M2), and SPP-HA D219/265A (mutant 3; M3) were infected with a lentivirus expressing FLAG-core (lower). (**j**) Huh7 and SPPKOHuh7 cells stably expressing SPP-HA (WT) and SPP-HA (M3) were infected with a lentivirus expressing FLAG-core. The data are representative of either three (**b**,**c**) or two (**e**–**j**) independent experiments.

γ-Secretase is a multisubunit protease complex that cleaves amyloid precursor proteins^21^. Its deregulation is associated with Alzheimer's disease. Because γ-secretase and SPP have similar enzymatic active sites for proteases, we first examined the effects of γ-secretase inhibitors in blocking the maturation of the HCV core protein via the inhibition of SPP activity^22^,^23^. Complementary DNA (cDNA) encoding a recombinant HCV core protein (1-191aa, genotype 1b) carrying FLAG (N terminus) and HA (C terminus) tags and possessing a mutation from Ala191 to Arg to inhibit cleavage by a signal peptidase^24^,^25^ along with green fluorescent protein (GFP; Fig. 1a) was expressed in Huh7 cells and incubated for 2 days in the presence of various concentrations of the inhibitors. None of the compounds exhibited toxicity to Huh7 cells in our assay (Supplementary Fig. 1a). Although only slight amounts of immature core protein (FLAG-core-HA) unprocessed by SPP were detected using anti-HA antibody, a significant and dose-dependent increase in the expression of the immature core protein was detected in cells treated with LY-411575 and RO4929097 but not in cells treated with the other inhibitors (Fig. 1b). In contrast, the expression of the mature HCV core protein (FLAG-core) that was detectable by anti-FLAG antibody was reduced in cells treated with LY-411575 and RO4929097 in a dose-dependent manner (Fig. 1b). GFP expression was not affected by the inhibitor treatments. The anti-HA antibody we used (Roche, anti-HA high affinity, clone 3F10) possessed a high affinity to the antigen; therefore, we could detect unprocessed core protein when using this antibody but not when using the anti-FLAG antibody. These results suggest that LY-411575 and RO4929097 have inhibitory activity against SPP and that the inhibition of SPP activity leads to the suppression of mature HCV core protein expression.

Next, we generated SPP-deficient (SPP^−/−^) mouse embryonic fibroblasts (MEFs) from SPP gene-targeted mice (Supplementary Fig. 1a,c). Although embryos from the SPP^−/−^ mice were smaller than those from control mice and died after embryonic day 13.5 (E13.5) (Fig. 1c,d), histological analysis revealed no obvious abnormalities in the embryonic structures (data not shown). The immature core protein was clearly detected in SPP^−/−^ MEFs, but only a small amount was present in wild-type MEFs (Fig. 1e), while the mature core protein was detected only in the wild-type MEFs. When the core and E1 proteins were expressed as a polyprotein in SPP^−/−^ MEFs, core protein expression was reduced, but E1 expression was not (Fig. 1f), suggesting that processing by SPP is crucial for the stable expression of its substrates.

### SPP substrate is expressed in a peptidase-dependent manner

A previous study showed that SPP cleaves the core protein of EHcV, but not that of Japanese encephalitis virus (JEV)^5^; therefore, we investigated the expression of the core proteins of EHcV and JEV in SPP^−/−^ MEFs. The expression of the EHcV core protein was significantly suppressed in SPP^−/−^ MEFs but not in wild-type MEFs (Fig. 1g), in contrast to the JEV core protein, which exhibited no reduction in SPP^−/−^ MEFs (Fig. 1h).

SPP has two putative protease active sites at Asp219 and Asp265 (Fig. 1i)^1^. HCV core protein expression was recovered in SPP^−/−^ MEFs by expression of the wild-type SPP, but not by the expression of loss-of-function mutants (Fig. 1i; Supplementary Fig. 1c). We generated SPP-deficient Huh7 (SPPKO Huh7) cells using a CRISPR/Cas9 system (Supplementary Fig. 1d,e) and found that HCV core protein expression was reduced and could be recovered by expressing the wild-type SPP, but not the M3 mutant (Fig. 1j). These results suggest that the expression of the HCV core protein and other SPP substrates can be suppressed in an SPP-dependent manner.

### Immature core protein is rapidly degraded by proteasome

**Figure 2:**
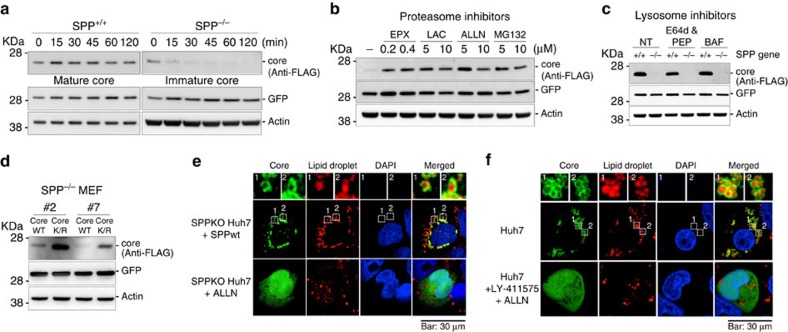
Ubiquitin-proteasomal degradation of the immature HCV core protein in SPPKO cells. (**a**) SPP^+/+^ MEFs and SPP^−/−^ MEFs infected with a lentivirus expressing FLAG-core were treated with cycloheximide (100 μM) at 48 h post infection and were subjected to immunoblotting at the indicated time points. (**b**) SPP^−/−^ MEFs infected with a lentivirus expressing FLAG-core were treated with proteasome inhibitors (epoxomicin: EPX; lactacystin: LAC, ALLN and MG132) at 36 h post infection. (**c**) SPP^+/+^ and SPP^−/−^ MEFs expressing FLAG-HCV core expressed by lentivirus were treated with E-64d (30 μM)/pepstatin A (1.5 μΜ; E64d and PEP) or bafilomycin (BAF; 10 nM) for 8 h at 48 h post infection and were subjected to immunoblotting. (**d**) SPP^−/−^ MEFs infected with lentivirus expressing FLAG-HCV core (Core) or its mutant with all lysine residues replaced with arginine residues (Core K/R) were subjected to immunoblotting at 48 h post infection. (**e**) Localization of the HCV core in SPPKO Huh7 cells. SPPKO Huh7 cells (bottom) and SPPKO Huh7 cells stably expressing SPP-HA (top) were transfected with pCAG OSF-HCV core and incubated for 48 h. To visualize the HCV core protein in SPPKO Huh7 cells, a proteasome inhibitor (ALLN) was administered for 10 h. The cells were fixed, and FLAG-HCV core (green), lipid droplets (red) and nuclei (blue) were stained with anti-FLAG antibody, HCS LipidTOX Red (Life Technologies) and DAPI, respectively. Scale bar, 30 μm. (**f**) Localization of the HCV core in cells treated with LY-411575. Huh7 cells transfected with pCAG OSF-HCV core were treated with LY-411575 and ALLN for 10 h at 48 h post transfection. Scale bar, 30 μm. The data are representative of either three (**a**,**b**) or two (**c**–**f**) independent experiments

We next examined the stability of the HCV core protein in cells treated with cycloheximide, a protein synthesis inhibitor. The mature HCV core protein produced in wild-type MEFs was stable over 120 min; in contrast, the immature HCV core protein was rapidly degraded in SPP^−/−^ MEFs within 15 min (Fig. 2a). Next, the HCV core protein was expressed in SPP^−/−^ MEFs and treated with various inhibitors of the proteasome and lysosome. The expression of the HCV core protein in SPP^−/−^ MEFs was significantly restored by treatment with proteasomal inhibitors (epoxomicin, lactacystin, Ac–Leu–Leu–Nle–Aldehyde (ALLN) and MG-132), while no effect was observed with lysosomal inhibitors (E-64d/pepstatinA and bafilomycin; Fig. 2b,c). The reduction in the HCV core protein by treatment with LY-411575 in Huh7 cells was restored when ALLN, a proteasome inhibitor, was present (Supplementary Fig. 2). Next, we designed an HCV core construct that replaced all Lys residues with Arg (Core K/R)^26^ and expressed it in SPP^−/−^ MEFs. Core K/R expression in SPP^−/−^ MEFs was significantly higher than that of the wild-type core protein (Fig. 2d), suggesting that ubiquitination is required for the degradation of the HCV core protein in SPP^−/−^ MEFs. The HCV core protein was mainly detected around lipid droplets in SPPKO Huh7 cells with restored SPP expression, as previously reported in Huh7 cells^27^ (Fig. 2e, top), whereas co-localization of the core protein with lipid droplet disappeared in SPPKO Huh7 cells (Fig. 2e, bottom). In addition, the treatment of Huh7 cells with LY-411575 also disrupted the co-localization of core protein with lipid droplets (Fig. 2f). Taken together, these results suggest that the immature HCV core protein unprocessed by SPP is quickly degraded via a ubiquitin/proteasome-dependent pathway.

### An SPP inhibitor ameliorates liver failure in HCV CoreTg mice

**Figure 3:**
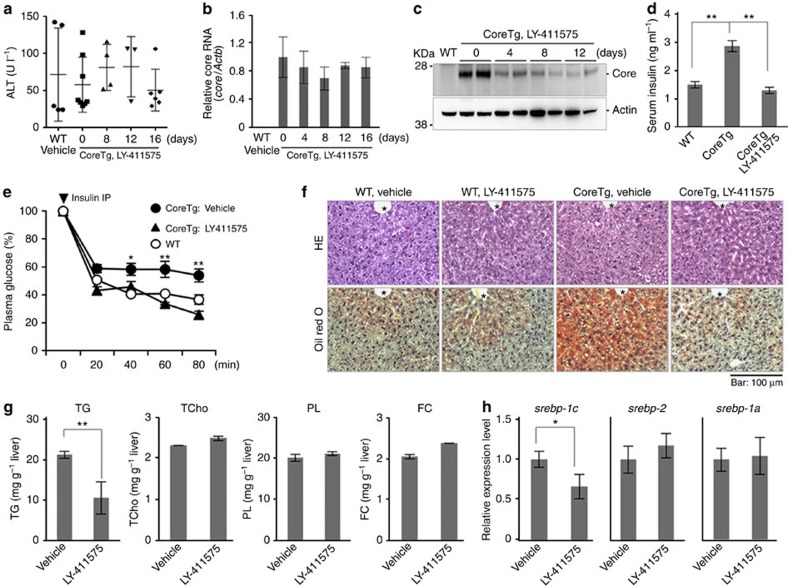
Amelioration of insulin resistance and steatosis in CoreTg mice by treatment with an SPP inhibitor. (**a**) CoreTg mice (male, 16 weeks old, *n*=3–10 in each group) were orally administered LY-411575 (10 mg kg^−1^) once a day. The ALT levels in the sera of mock-treated WT and CoreTg mice treated with LY-411575 were determined. (**b**) Core mRNA expression was determined by qPCR. (**c**) The livers of CoreTg mice treated with LY-411575 as described above were collected at each time point and subjected to immunoblotting. (**d**) Serum insulin concentrations of CoreTg mice (*n*=4) treated with or without LY-411575 for 16 days under a fed condition. Significant differences are indicated by double asterisks (**P<0.01). (**e**) CoreTg mice were orally administered LY-411575 or vehicle once a day for 16 days and fasted during the test period and then intraperitoneally injected with human insulin (1.5 U kg^−1^). Plasma glucose levels were determined after normalization to the baseline glucose concentration at the time of insulin administration (arrowhead). Significant reductions (*P<0.05, **P<0.01) in the plasma glucose levels were observed in CoreTg mice in response to treatment with LY-411575. (**f**) CoreTg and WT mice (24 weeks old) were orally administered LY-411575 or vehicle once a day for 16 days. Liver sections were collected post administration and stained with HE (upper) and Oil Red O (lower). Scale bar, 100 μm. *: Central vein. (**g**) The amounts of TG, Tcho, PL and FC in the liver collected post administration (*n*=3 in each group) were determined. A significant reduction (**P<0.01) in the TG concentration was observed in CoreTg mice in response to treatment with LY-411575. (**h**) SREBP-1c, SREBP-2 and SREBP-1a expression levels in the livers were determined by qPCR. A significant reduction (*P<0.05) in SREBP-1c expression was observed in CoreTg mice in response to LY-411575 treatment. The data represent the mean±s.e. from two independent measurements of at least three mice per genotype, time point and compound treatment. The significance of the differences was determined using Student's *t*-test. Images are representative of at least three independent mice per genotype and compound treatment.

We next examined the effect of LY-411575 on liver pathogenesis in CoreTg mice. No toxicity, including either alanine aminotransferase (ALT) elevation or weight loss, was observed in CoreTg mice after oral administration of LY-411575 for 16 days (Fig. 3a; Supplementary Fig. 3a). Although the messenger RNA (mRNA) expression of the HCV core protein in the livers of CoreTg mice was not changed by treatment with LY-411575 (Fig. 3b), the expression of the core protein was significantly reduced by this treatment (Fig. 3c). In addition, the high levels of serum insulin and insulin resistance observed in CoreTg mice^17^ were clearly improved by the administration of LY-411575 (Fig. 3d,e). Although no remarkable differences were observed by haematoxylin and eosin (HE) staining, Oil Red O staining of the liver sections revealed that the lipid accumulation in CoreTg mice was also ameliorated by treatment with LY-411575 for 16 days at the age of 24 weeks (Fig. 3f). The quantities of triglycerides (TG), but not of total cholesterol (TCho), phospholipids (PL) or free cholesterol (FC), in the livers of CoreTg mice were decreased by the treatment with LY-411575 (Fig. 3g). The quantity of liver fatty acids is tightly controlled by SREBP-1c, a family of steroid regulatory element-binding proteins (SREBPs)^28^,^29^. SREBP-1c mRNA expression, but not SREBP-1a or SREBP-2 expression, was reduced in the livers of CoreTg mice on treatment with LY-411575 (Fig. 3h). Moreover, we confirmed that treatment with LY-411575 suppressed the levels of serum amyloid-β peptides (Aβ40 and Αβ42), which are cleavage products of γ-secretase (Supplementary Fig. 3b,c). LY-411575 was originally characterized as a γ-secretase inhibitor. Because LY-411575 inhibits not only γ-secretase and Notch signalling but also SPP, it is unclear which inhibitory effects of LY-411575 participate in the improvement of CoreTg-induced insulin resistance and steatosis. To examine the roles of SPP in the pathogenesis in CoreTg mice, we used SPP^+/−^ mice.

### SPP haploinsufficiency improves liver steatosis in CoreTg mice

**Figure 4:**
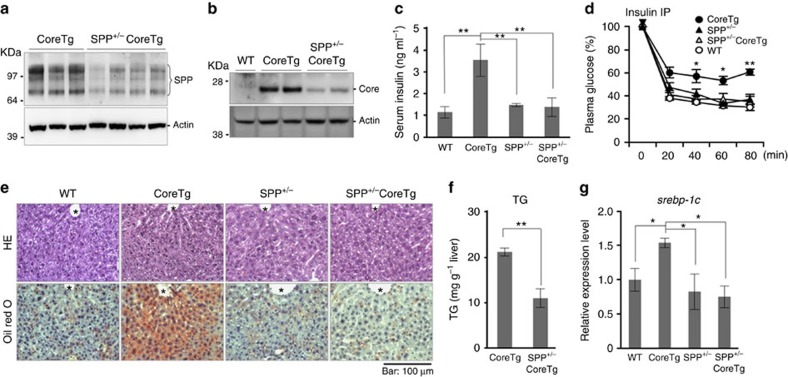
SPP haploinsufficiency does not cause insulin resistance or steatosis in CoreTg mice. (**a**) Liver lysates from CoreTg male mice (*n*=3) and SPP^+/−^CoreTg male mice (*n*=4) were subjected to immunoblotting using anti-SPP and anti-Actin antibodies. (**b**) Liver lysates from male mice of the WT, CoreTg and SPP^+/−^CoreTg groups were subjected to immunoblotting. (**c**) The serum insulin concentrations of four mice of each of the WT, CoreTg, SPP^+/−^ and SPP^+/−^CoreTg groups (18–19 weeks old) under fed conditions were determined by ELISA. Significant increases are indicated by double asterisks (**P<0.01). (**d**) WT (open circles), CoreTg (closed circles), SPP^+/−^ (closed triangle) and SPP^+/−^CoreTg (open triangles) mice (13–16 weeks old, *n*=7 in each group) were fasted during the test period and intraperitoneally injected with human insulin (1.5 U kg^−1^). The plasma glucose levels were determined at the indicated time points after normalization to the baseline glucose concentration at the time of insulin administration (arrowhead). Significant reductions in the plasma glucose levels in SPP^+/−^CoreTg mice compared with those in CoreTg mice are indicated by asterisks (*P<0.05, **P<0.01). (**e**) Livers were collected from WT, CoreTg, SPP^+/−^ and SPP^+/−^CoreTg mice (24 weeks old), and liver sections were stained with HE (upper) and Oil Red O (lower). Scale bar, 100 μm. *: Central vein. (**f**) Amounts of TG in the liver of CoreTg and SPP^+/−^CoreTg mice (24–26 weeks old, *n*=3 in each genotype) were determined. A significant reduction (**P<0.01) in the TG concentration compared to CoreTg mice was observed in SPP^+/−^CoreTg mice. (**g**) Total RNA was prepared from the livers of WT, CoreTg, SPP^+/−^ and SPP^+/−^CoreTg mice (24–32 weeks old, male, *n*=4–6 in each genotype), and SREBP-1c expression levels were determined by qPCR. A significant reduction (*P<0.05) in SREBP-1c expression was observed with SPP haploinsufficiency in CoreTg mice. The data represent the mean±s.e. from two independent measurements of at least three mice per genotype and time point. The significance of the differences was determined using Student's *t*-test. Images are representative of at least three independent mice per genotype.

Next, we generated SPP^+/−^CoreTg mice to examine the effects of SPP haploinsufficiency on liver pathogenesis induced by the HCV core protein. Although SPP^+/−^CoreTg mice expressed less SPP mRNA and protein in the liver compared to CoreTg mice (Fig. 4a; Supplementary Fig. 4a), no significant abnormalities in development or body weight (Supplementary Fig. 4b) were observed. Notably, we found that the expression of the HCV core protein in SPP^+/−^CoreTg mice was significantly lower than that in CoreTg mice, even though comparable amounts of core protein mRNA were detected (Fig. 4b; Supplementary Fig. 4c). The high levels of serum insulin in CoreTg mice were reduced in SPP^+/−^CoreTg mice, even compared with the normal levels observed in control mice (Fig. 4c). In addition, an insulin resistance test revealed that the insulin resistance state observed in CoreTg mice was recovered in SPP^+/−^CoreTg mice (Fig. 4d). Whereas HE staining revealed no significant lesions, Oil Red O staining of the liver sections revealed the accumulation of lipid droplets characteristic of steatosis in CoreTg mice, while no lipids were accumulated in the wild-type, SPP^+/−^ or SPP^+/−^CoreTg mice (Fig. 4e). The total amounts of TG, but not Tcho, PL and FC, were lower in the livers of SPP^+/−^CoreTg mice compared with CoreTg mice (Fig. 4f; Supplementary Fig. 4d). The enhanced level of SREBP-1c mRNA expression was significantly reduced in the livers of SPP^+/−^CoreTg mice compared with the normal level observed in wild-type mice, but the levels of SREBP-1a and SREBP-2 mRNA expression were unchanged (Fig. 4g; Supplementary Fig. 4e). To exclude the possibility that SPP affects the induction of liver steatosis independent of the HCV core protein, wild-type, CoreTg, SPP^+/−^ and SPP^+/−^CoreTg mice were fed a choline-deficient L-amino-acid-defined (CDAA) diet to promote non-alcoholic steatosis for 4 weeks, and all of the mice developed diet-induced liver steatosis (Supplementary Fig. 4f). To evaluate the involvement of γ-secretase activity in SPP haploinsufficiency, the serum production levels of Aβ40 and Αβ42 were determined in CoreTg and SPP^+/−^CoreTg mice. No significant difference in protein production was observed between CoreTg and SPP^+/−^CoreTg mice (Supplementary Fig. 4g), suggesting that SPP, but not γ-secretase, activity participates in HCV-induced pathogenesis. These results support the theory that SPP haploinsufficiency in CoreTg mice was recovered from liver steatosis through the repression of HCV core protein expression.

### SPP is an essential host factor for HCV propagation

**Figure 5:**
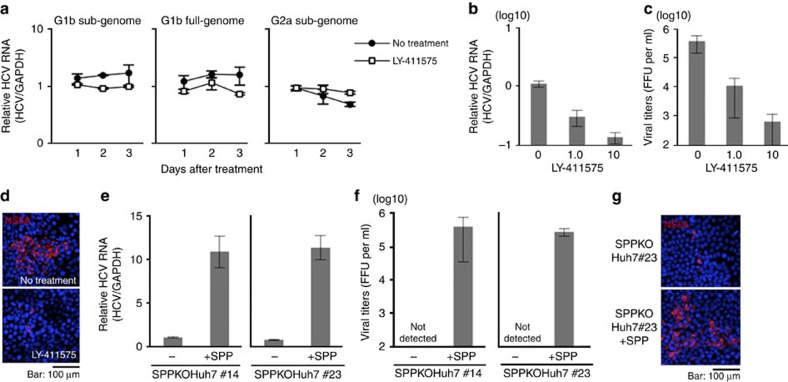
SPP is an essential host factor for HCV propagation. (**a**) Sub- and full-genomic genotype 1b replicon (left and middle) and sub-genomic genotype 2a replicon (right) cells were treated with LY-411575 (1 μM) for 3 days. The amounts of HCV RNA were determined by qPCR. The data represent the mean±s.d. of 2 experiments performed with a cell line representative of each replicon cell line. (**b**,**c**) Huh7 cells infected with the HCV JFH-1 strain at a multiplicity of infection (moi) of 0.5 were treated with LY-411575 (1 or 10 μM) for 4 days. HCV propagation was evaluated by qPCR analysis for intracellular HCV RNA. (**b**). Infectious titres in the culture supernatants were determined using a focus-forming assay (**c**). The data represent the mean±s.d. of two independent experiments. (**d**) Huh7 cells infected with the HCV JFH-1strain at a moi of 0.5 were overlaid with methylcellulose in the presence or absence of LY-411575 (1 μM) for 3 days. The cells were fixed, and NS5A (red) and nuclei (blue) were stained with anti-HCV NS5A antibody and DAPI, respectively. Scale bar, 100 μm. Images are representative of two independent experiments. (**e**,**f**) Two SPPKOHuh7 cell lines (#14 and #23) were infected with the HCV JFH-1strain at an moi of 0.5, and intracellular HCV RNA and infectious titres in the culture supernatants were determined by qPCR (**e**) and focus-forming assay (**f**) at 4 days post infection, respectively. The data represent the mean±s.d. of two independent experiments performed with a cell line representative of SPPKOHuh7 cells. (**g**) SPPKOHuh7 cells (clone #14) and those with restored SPP expression were infected with the HCV JFH-1 strain at an moi of 0.5, and NS5A (red) and nuclei (blue) were stained with anti-HCV NS5A antibody and DAPI, respectively, at 3 days post infection. Scale bar, 100 μm. Images are representative of two independent experiments.

To determine the roles of SPP in HCV RNA replication, HCV replicon cells were treated with LY-411575. However, no significant effect was observed on the viral replication of the genotype 1b sub- and full-genomic replicon (Con1 strain) or genotype 2a (JFH-1 strain) sub-genomic replicon (Fig. 5a)^30^,^31^,^32^. Next, to examine the effects of LY-411575 on infectious particle production, HCV (JFH-1 strain) was inoculated into Huh7 cells in the presence of LY-411575. The total amounts of HCV RNA (Fig. 5b) and infectious particles in the supernatants (Fig. 5c) were dose-dependently decreased with LY-411575 treatment, and a focus assay also showed that LY-411575 inhibited viral spread (Fig. 5d). However, semagacestat, which lacks an inhibitory effect on the processing of HCV core protein (Fig. 1b), exhibited no effect on the production of infectious particles (Supplementary Fig. 5a,b). In addition, the replication of total intracellular HCV RNA and the formation of infectious particles in the supernatants were severely impaired in SPPKOHuh7 cells, whereas the complementation of SPP in SPPKOHuh7 cells restored HCV propagation, including intracellular HCV RNA, infectious particle formation in the supernatant, and focus formation (Fig. 5e–g). Moreover, SPP expression had no significant effect on JEV or Dengue virus (DENV) propagation in Huh7 cells (Supplementary Fig. 5c). Furthermore, no significant difference was observed in the expression of interferon-stimulated gene 15 (ISG15) in either SPPKO or SPP-restored cells infected with either vesicular stomatitis virus (VSV) or HCV (Supplementary Fig. 5d). These results suggest that SPP plays a crucial role in HCV propagation.

### TRC8 is required for degradation of the immature core protein

**Figure 6:**
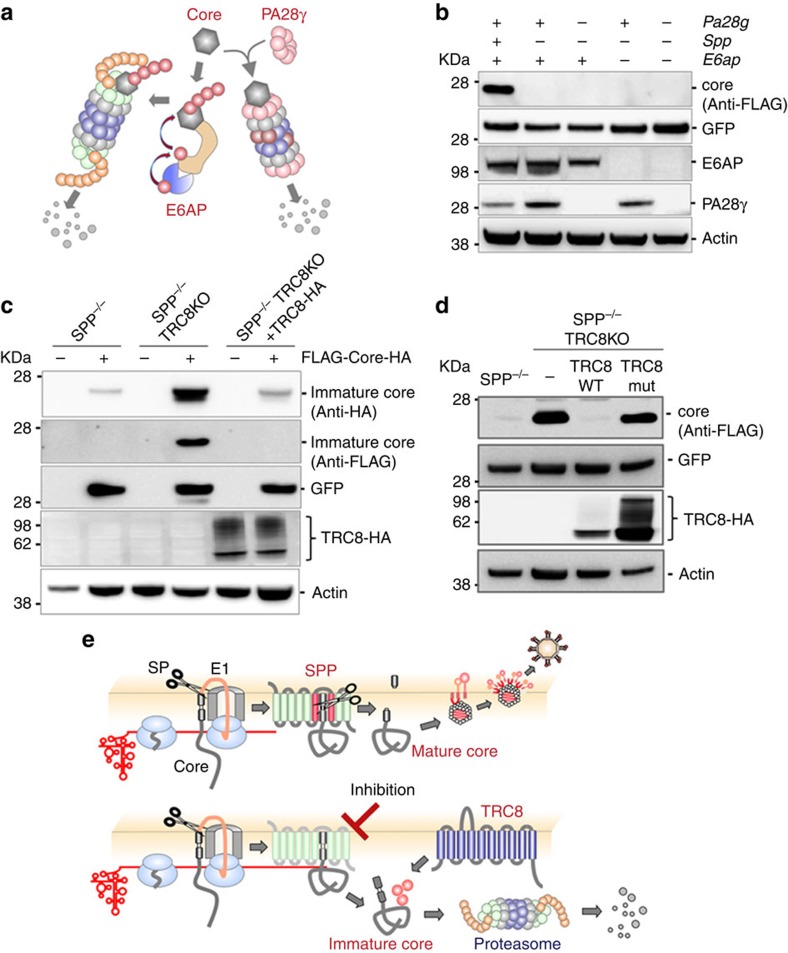
TRC8-dependent proteasome degradation of the immature core protein in SPP-deficient cells. (**a**) Schematic representations of two distinct pathways of HCV core protein degradation. The mature core protein interacts with PA28γ in the nucleus and is degraded by the proteasome independent of ubiquitin^52^. In contrast, E6AP can also bind to the mature core protein in the perinuclear region. E6AP ubiquitinates the core protein and degrades it in an ubiquitin-dependent manner^34^. (**b**) SPP/PA28γ/E6AP triple-knockout MEFs (SPP/PA28γ/E6APTKO) expressing FLAG-HCV core via lentivirus were subjected to immunoblotting at 48 h post infection. (**c**) SPP^−/−^ MEFs, SPP^−/−^TRC8KO MEFs and SPP^−/−^TRC8KO MEFs restored by HA-tagged TRC8 expression were infected with a lentivirus expressing FLAG-core-HA and subjected to immunoblotting. (**d**) SPP^−/−^ and SPP^−/−^TRC8KO MEFs complemented with TRC8-HA or TRC8mut-HA and expressing FLAG-core were subjected to immunoblotting. (**e**) Maturation of the HCV core protein by processing with SP and SPP (upper panel). SPP deficiency and treatment with an inhibitor for SPP induced the degradation of the immature core protein by the proteasome, mediated by the E3 ligase TRC8 (lower scheme). The data are representative of two independent experiments.

Previous studies demonstrated two distinct proteasome-dependent degradation pathways for the HCV core protein (Fig. 6a). First, the proteasome activator 28γ (PA28γ) was shown to interact with the HCV core protein in the nucleus and to cause proteasomal degradation in an ATP/ubiquitin-independent manner^33^. Second, E6-associated protein (E6AP), an E3 ubiquitin ligase, also associated with the HCV core protein in the cytoplasm and induced degradation in a ubiquitin-dependent manner^34^. Therefore, we established SPP/PA28γ/E6AP triple-knockout (TKO) MEFs using a CRISPR/Cas9 system. Expression of the HCV core protein was not restored in the SPP/PA28γ/E6APTKO MEFs (Fig. 6b). Furthermore, LY-411575 treatment still induced the degradation of the HCV core protein in the TKOMEFs complemented with SPP (Supplementary Fig. 6a). These results suggest that neither PA28γ nor E6AP is involved in immature HCV core protein degradation.

Recent studies have revealed that SPP participates in ERAD^8^,^10^,^35^. To identify an E3 ligase responsible for the degradation of the immature core protein, we performed an siRNA-mediated screening of ERAD-associated E3 ligases and found that knockdown of TRC8 inhibits the degradation of the HCV core protein (Supplementary Fig. 6b,c). Knockout of TRC8 significantly inhibited the degradation of the immature HCV core protein in SPP^−/−^ MEFs, and complementation of TRC8 in SPP/TRC8DKO MEFs recovered the degradation of the immature HCV core protein (Fig. 6c; Supplementary Fig. 6d). Mutations of Cys447 and Cys450 in TRC8 abolish the E3 ubiquitin ligase activity^36^. A TRC8 mutant in which Cys447 and Cys450 were substituted to Ala was expressed in SPP/TRC8DKO cells. The immature core protein was not degraded in DKO cells expressing the mutant TRC8 (Fig. 6d), suggesting that the E3 ubiquitin ligase activity of TRC8 is required for immature HCV core protein degradation. In addition, the complementation of SPP in the SPP/TRC8 DKO MEFs partially inhibited LY-411575-induced degradation of the HCV core protein (Supplementary Fig. 6e). Furthermore, the TRC8 mutant interacted with immature core proteins in SPP-deficient cells (Supplementary Fig. 6f). Next, to determine the role of the ERAD factor Derlin-1 on the degradation of the immature HCV core protein, SPP and Derlin-1 DKO MEFs were established, but no significant effect was observed (Supplementary Fig. 6g). Moreover, the siRNA-mediated knockdown of not only Derlin-1 but also Derlin-2 resulted in no apparent change in the expression of the immature HCV core protein (Supplementary Fig. 6h). Although we cannot exclude the possibility of the involvement of other E3 ligases, these results suggest that TRC8 is a major E3 ligase that participates in the degradation of the immature HCV core protein generated in cells lacking SPP activity or treated with an SPP inhibitor (Fig. 6e).

### Expression of the immature HCV core protein induces ER stress

**Figure 7:**
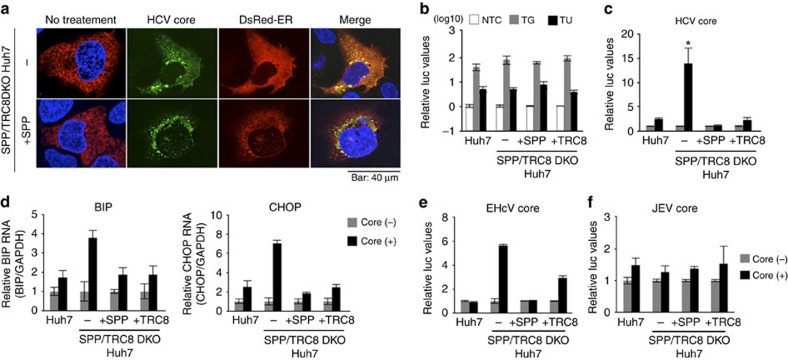
The HCV core altered the ER distribution and induced ER stress in SPP/TRC8DKO Huh7 cells. (**a**) SPP/TRC8DKO Huh7 cells (bottom) and those stably expressing SPP-HA (top) were transfected with pCAG OSF-HCV core and pCMV DsRed-ER and incubated for 48 h. FLAG-HCV core (green) and nuclei (blue) were stained with anti-FLAG antibody and DAPI, respectively. Scale bar, 40 μm. Images are representative of two independent experiments. (**b**) The luciferase activity was determined in cells transfected with a reporter plasmid containing spliced XBP1 (ERAI) and pRL-SV40 and treated with 1 μM of thapsigargin (TG) or tunicamycin (TU) 24 h post transfection. The data represent the mean±s.d. of two independent experiments. (**c**) The luciferase activity was determined in cells transfected with plasmids encoding the HCV core, ERAI and pRL-SV40 at 24 h post transfection. Significant enhancement (*P<0.05) of XBP1s production was observed with HCV core expression only in SPP/TRC8DKO Huh7 cells. The data represent the mean±s.d. of two independent experiments performed with a cell line representative of Huh7 cells. (**d**) RNA was extracted from cells transfected with an HCV core protein expression plasmid of at 24 h post transfection, and binding of immunoglobulin protein (BIP; left) and CCAAT/enhancer-binding protein homologous protein (CHOP; right) expression levels were quantified by qPCR. The data represent the mean±s.d. of two independent experiments performed with a cell line representative of Huh7 cells. (**e**,**f**) The luciferase activity was measured in cells transfected with plasmids encoding the EHcV core (**e**) or JEV core (**f**), ERAI and pRL-SV40 at 24 h post transfection. The data represent the mean±s.d. of two independent experiments performed with a cell line representative of Huh7 cells. The significance of the differences was determined using Student's *t*-test.

Next, we established SPP/TRC8DKO Huh7 cells using a CRISPR/Cas9 system (Supplementary Fig. 7a,b,d). The degradation of the immature HCV core protein was impaired in SPP/TRC8DKO Huh7 cells, similar to that seen in SPP/TRC8DKO MEFs (Supplementary Fig. 7c,e). Although the HCV core proteins in SPP/TRC8DKO Huh7 cells with restored SPP expression were co-localized with lipid droplets, the proteins in SPP/TRC8DKO Huh7 cells exhibited ER localization (Fig. 7a). Notably, the ER was distributed throughout the cytoplasm in SPP/TRC8DKO Huh7 cells, but not in those with restored SPP expression. ER expansion, as seen in SPP/TRC8DKO Huh7 cells, was previously shown to be induced by ER stress through the expression of spliced X-box binding protein 1 (XBP1s)^37^,^38^,^39^. Therefore, we hypothesized that the expression of the immature HCV core protein in SPP/TRC8DKO cells induces ER stress via the expression of XBP1s. To investigate this hypothesis, the expression of XBP1s was measured by a reporter assay in SPP/TRC8DKO Huh7 cells by HCV core protein expression. Although the treatment of SPP/TRC8DKO Huh7 cells with either thapsigargin or tunicamycin, which are commonly used for the induction of ER stress, resulted in a clear enhancement of XBP1s compared to the non-treated control (Fig. 7b), HCV core protein expression significantly enhanced XBP1s expression in SPP/TRC8DKO cells, but not in cells with restored expression of either SPP or TRC8 (Fig. 7c), suggesting that XBP1s was induced by immature HCV core protein expression. HCV core protein expression in SPP/TRC8DKO Huh7 cells, but not in cells with restored expression of either SPP or TRC8, enhanced the mRNA levels of ER stress-inducible genes, binding of immunoglobulin protein (BIP) and CCAAT/enhancer-binding protein homologous protein (CHOP) (Fig. 7d). Furthermore, the expression of the core protein of EHcV, but not the core protein of JEV-enhanced reporter gene expression in SPP/TRC8DKO Huh7 cells, but not in cells restored with either SPP or TRC8 (Fig. 7e,f), suggesting that the immature core proteins of HCV and EHcV must be degraded to circumvent the undesired induction of ER stress. Our results highlight the role of the SPP/TRC8 axis in ER quality control for SPP substrates.

## Discussion

In this study, we evaluated the capacity of inhibitors of γ-secretase to prevent the maturation of the HCV core protein. Although several γ-secretase inhibitors^23^ were previously used as SPP inhibitors, the concentrations of LY-411575 and RO4929097, used to inhibit processing of the HCV core protein, were lower than 1 μΜ, in contrast to concentrations of more than 10 μΜ for other inhibitors^15^. LY-411575 and RO4929097 had more potent capacities to inhibit HCV core protein processing at lower concentrations. All of the γ-secretase inhibitors we tested in this study were well-established as non-transition-state analogues for γ-secretase, which interacted with its substrate-binding sites. Notably, semagacestat, which failed in phase III trials for the treatment of Alzheimer's disease^40^, did not exhibit any inhibitory effect on SPP activity in our assay, suggesting that the accessibilities of these compounds to γ-secretase and SPP are different due to the difference in their structures. Therefore, it might be feasible to develop compounds that specifically inhibit SPP activity.

We demonstrated that treatment with an SPP inhibitor and deficiency of SPP genes induced the degradation of the HCV core protein both *in vivo* and *in vitro*. Boname *et al*.^6^ established an SPP-deficient HCT116 cell line and identified Haem oxygenase-1 (HO-1) as an SPP substrate; however, their experiments did not clarify the mechanistic consequences of HO-1 degradation. Chen *et al*. reported that SPP formed a complex with the ERAD factor Derlin1 and TRC8, as shown by an immunoprecipitation assay^8^. We also identified TRC8 as an E3 ligase involved in the degradation of the immature HCV core protein, but our data suggest that the Derlin family members do not participate in the degradation of the immature HCV core protein. Although it is still unclear whether classical ERAD pathways are involved in the degradation of immature core protein, further studies are needed to clarify the precise molecular mechanisms of the degradation of SPP substrates.

The HCV core protein is a multifunctional protein that is required for the formation of infectious particles and is associated with liver diseases^14^. Previous studies demonstrated that cleavage of the HCV core protein by SPP is crucial for viral propagation^15^,^16^. Our present data clearly indicate that the immature HCV core protein is not able to produce infectious HCV particles. Therefore, we can conclude that processing of the HCV core protein by SPP is essential for the production of infectious HCV particles. In addition, the oral administration of LY-411575 and the haploinsufficiency of the SPP gene reduced the expression of the HCV core protein and ameliorated insulin resistance and liver steatosis in CoreTg mice. Indeed, LY-411575 was originally developed as an inhibitor of Notch signalling and γ-secretase, and treatment with LY-411575 resulted in reductions in the serum levels of Aβ40 and Aβ42 in both wild-type and CoreTg mice (Supplementary Fig. 4). No significant changes were observed in mice haploinsufficient for the SPP gene (Supplementary Fig. 5), suggesting that SPP activity, but not γ-secretase activity or Notch signalling, participates in the development of pathogenesis induced by the HCV core protein in CoreTg mice.

Recent studies have revealed the participation of SPP in ER quality control. A heavy chain molecule of major histocompatibility complex (MHC) class I was dislocated through its interaction with US2 and US11 encoded by human cytomegalovirus^35^, and US2 also induced the degradation of heavy chain molecules through its association with SPP and an ERAD-associated E3 ubiquitin ligase, TRC8 (refs 8, 10). In contrast, US2 still caused the degradation of MHC class I molecules in SPP^−/−^ HCT116 cells^6^. Our results illustrate that the inhibition of SPP activity induced the production of the immature HCV core protein, which was quickly degraded by a TRC8-dependent proteasome pathway, but that knockout of the SPP and/or TRC8 genes exhibited no effect on the induction of ER stress in cells treated with either thapsigargin or tunicamycin, suggesting that neither SPP nor TRC8 is involved in the induction of a canonical ER stress response. Furthermore, the immature HCV core protein produced in cells with knockout of both the SPP and TRC8 genes was retained in the ER, where it induced XBP1s through the induction of ER stress and the accumulation of phospholipids in the cytosol, which finally induced ER expansion^38^. ER retention of the immature HCV core protein in SPP/TRC8DKO Huh7 cells was disrupted by the expression of TRC8 (Figs 2e and 7a), suggesting that recognition by TRC8 is required for the translocation of the immature HCV core protein to be degraded by the proteasome. Moreover, it has been shown that the hepatocytes of hepatitis C patients exhibit ER stress^41^. Collectively, these data suggest that SPP and TRC8 play a redundant role in the circumvention of the ER stress induced by the immature HCV core protein and also participate in the induction of ER stress *in vivo*.

TRC8 was originally identified as a translocated gene in a family with hereditary renal cell carcinoma^42^. TRC8 possesses a sterol-sensing domain that is capable of interacting with a sterol. This domain is also detected in 3-hydroxyl-3-methylglutaryl coenzyme A (HMG-CoA) reductase, the SREBP cleavage-activating protein (SCAP), the Niemann–Pick C1 protein and Patched^43^. TRC8 has been proposed to regulate the activity of SREBP-2, a major regulator of cholesterol metabolism, through its interaction with the SREBP-2/SCAP complex^44^. Another study reported that TRC8 formed a complex with gp78 and degraded HMG-CoA reductase^45^. Disorders of lipid metabolism, such as obesity, insulin resistance and type 2 diabetes, are often associated with non-alcoholic fatty liver disease and hepatocellular carcinoma^46^. A recent report suggested that TRC8 is a tumour suppressor^7^ and that B cells derived from XBP1KO mice exhibited downregulation of SPP^37^. Analysis of knockout mice deficient in the TRC8 and/or SPP genes might clarify the biological significance of the interaction between SPP and TRC8 in HCV pathogenesis.

In conclusion, we demonstrated that the maturation of the HCV core protein by SPP is crucial for the production of infectious HCV particles and for the induction of hepatic diseases, including insulin resistance and steatosis, in CoreTg mice. The immature HCV core protein generated by the inhibition of SPP activity induced ER stress but was degraded by the proteasomal pathway through TRC8, suggesting that both SPP and TRC8 participate in HCV propagation and pathogenesis. Although further studies are needed to clarify the biological significance of degradation of the HCV core protein in the HCV lifecycle in greater detail, SPP might serve as a novel drug target for chronic hepatitis C through the inhibition of HCV core protein maturation.

## Methods

### Viruses and cell lines

HEK293T, and Huh7, Huh7.5.1 and Vero cells were obtained from the National Institute of Infectious Diseases (Japan) and were cultured in DMEM supplemented with 10% fetal bovine serum, 100 U ml^−1^ penicillin and 100 μg ml^−1^ streptomycin. Replicon cells harbouring genotype 1b (Con1) or 2a (JFH-1) of the HCV genome were maintained in DMEM supplemented with 10% fetal bovine serum, 1 μg ml^−1^ G418, 100 U ml^−1^ penicillin and 100 μg ml^−1^ streptomycin. HCV derived from the genotype 2a JFH-1 strain mutated in E2, p7 and NS2 as previously shown^47^ was prepared by serial passages in Huh7.5.1 cells. JEV (AT31 strain^48^) and DENV (H241 strain^49^) were prepared in C6/36 cells. The infectious titres of HCV, JEV and DENV were determined using a focus-forming assay. Mouse monoclonal antibodies against HCV NS5A (5A27)^15^ and JEV/DENV NS3 (#1791)^50^ were used to visualize their focuses.

### Mice and preparation of MEFs

SPP^+/−^ mice were generated at Ozgene (Perth, Australia) by crossing SPP^fl/+^ with pGK-cre mice. SPP^+/−^ mice were crossed with CoreTg mice to generate SPP^+/−^CoreTg mice. The animals were maintained under a 12-h light/dark cycle (lights on at 08:00 hours) at room temperature (23 °C±2 °C). All animal experiments conformed to the Guidelines for the Care and Use of Laboratory Animals and were approved by the Institutional Committee of Laboratory Animal Experimentation (Research Institute for Microbial Diseases, Osaka University). All efforts were made to minimize animal suffering and to reduce the number of animals used in the experiments. SPP^−/−^ MEFs were generated by crossing SPP^+/−^ female and male mice, and pregnant female mice were euthanized on E13.5. Primary MEFs were seeded in a collagen-coated T25 flask (one per embryo). After a few days, confluent MEFs were passaged and immortalized using a lentivirus carrying the SV40 large T antigen.

### Antibodies and reagents

The following antibodies were obtained: anti-HCV NS5A mouse monoclonal antibody (5A27)^15^, anti-HCV core mouse monoclonal antibody (Fujirebio, Japan), anti-actin mouse monoclonal antibody (Sigma, A2228), anti-HA rat monoclonal antibody (Roche, clone 3F10), anti-GFP mouse monoclonal antibody (Clontech, JL-8), anti-E1 mouse monoclonal antibody (toru0726cb-1)^51^, anti-E6AP antibody (Sigma), anti-PA28γ antibody^52^, anti-p62 antibody (MBL) and horseradish peroxidase-conjugated anti-FLAG mouse monoclonal antibody (Sigma, clone M2). Anti-SPP rabbit polyclonal antibody was generated by immunization with synthetic peptides from amino acids 356 to 366 of mouse SPP (YEESNPKDPAA). LY-411575, ALLN (A6185), MG132, and bafilomycin A1 (B1793) were obtained from Sigma. RO4929097, avagacestat, semagacestat, DAPT (GSI-IX), and MK-0752 were purchased from Selleck Chemicals. Epoxomicin (4381-v), lactacystin (4368-v), E-64d (4321-v), and pepstatin A (4397-v) were from Peptide Institute, Inc.

### Plasmids and generation of lentiviruses

The lentiviral transfer vectors used in this study were generated from FUGW (Addgene, #14883). The PCR product of the internal ribosomal entry site fused with either GFP (primers; #349/#347) or puromycin and the *N*-acetyl-transferase gene (primers #165/#166) was cloned into FUGW digested by BamHI and EcoRI. The resulting plasmids were named FUIGW and FUIPW, respectively. The cDNAs for SPP (primers #1089/#1090) and TRC8 (primers #1621/#1624) were amplified and cloned into FUIPW digested by EcoRI. FLAG-tagged HCV core (primers #1439/1516), JEV core (primers #1439/#1381) and EcHV core (primers #1439/#951) were amplified and cloned into FUIGW digested by BamHI. FLAG-core-HA (primers #1439/#1440) and FLAG-core-E1 (primers #1439/#1578) were amplified and cloned into FUIGW digested by BamHI. To generate TRC8mut (C447/450A), two PCR fragments (primers #1621/#1622 and #1624/#1623) were amplified and cloned into FUIPW digested by EcoRI using the In-Fusion HD cloning kit (Clontech). For non-viral transfection, One-Strep tag and FLAG (OSF, primers #1110/#1111) and the HCV core (primers #1106/#1107) were amplified and cloned into pCAGGS^53^. The In-Fusion HD cloning kit (Clontech) was used for all plasmid constructions. The pCAG-VSV-G^54^ and pCMV-dR8.2 (Addgene, #12263) plasmids were used for generating lentiviruses. pCAG FLAG-JEV core^55^ and pcDNA3.1 Flag-EHcV core-HA^5^ were used in this study. Calreticulin genes encoding ER-targeting and ER-retrieval sequences were fused with DsRed at the N and C termini, respectively^25^. The ER-stress indicator (ERAI) and pRL-SV40 were purchased from TransGenic Inc. and Promega, respectively. All primers are shown in Supplementary Table 1. The plasmids used in this study were confirmed by sequencing with an ABI Prism 3130 genetic analyzer (Applied Biosystems).

To generate lentiviruses, HEK293T cells were transfected with a transfer vector, pCMV δR8.2 and pCMV-VSV-G, using polyethylenimine (PEI, Polysciences Inc., MW 25,000), and the culture supernatants collected at 3 days post transfection were passed through a 0.45-μm filtre. Culture supernatants containing the lentiviruses were inoculated into target cells seeded on six-well plates using Polybrene (Sigma) and were centrifuged at 2,500 r.p.m. for 45 min at 32 °C.

### Establishment of gene-knockout cell lines using a CRISPR/Cas9 system

The plasmids of pX330 (#42230) and pCAG EGxxFP (#50716) were obtained from Addgene. The target sequences from each gene are summarized in Supplementary Table 2. Genomic DNAs of Huh7 cells and MEFs were amplified and cloned into pCAG EGxxFP. Gene-knockout Huh7 cells and MEFs were constructed using a CRISPR/Cas9 system, as previously described^56^,^57^. Briefly, Huh7 cells were transfected with pX330 and pCAG EGxxFP and were incubated for 1 week. GFP-positive Huh7 cells were sorted by FACS and formed single colonies. Gene deficiency was confirmed by sequencing and western blotting.

### siRNA

Predesigned siRNAs (silencer select siRNA) against ERAD-related E3 ligases or other siRNAs were purchased from Life Technologies. Ten nanomoles of siRNA was transfected into Huh7-based cell lines using RNAiMAX transfection reagents (Life Technologies). The target sequence of each siRNA is summarized in Supplementary Table 3. The mRNA-knockdown efficiency was determined by quantitative RT-PCR (qPCR). The primer pairs used in the qPCR are summarized in Supplementary Table 4.

### qPCR

HCV RNA was quantified using a TaqMan RNA-to-Ct 1-Step Kit and the ViiA7 real-time PCR system (Life Technologies). The following primers were used: for HCV, 5′- GAGTGTCGTGCAGCCTCCA -3′ and 5′- CACTCGCAAGCACCCTATCA -3′; and for GAPDH, 5′- TGTAGTTGAGGTCAATGAAGGG -3′ and 5′- ACATCGCTCAGACACCATG -3′. The following probes were used: for HCV, 5′-6- FAM/CTGCGGAAC/ZEN/CGGTGAGTACAC/ -3′IABkFQ; and for GAPDH, 5′-6- FAM/AAGGTCGGA/ZEN/GTCAACGGATTTGGTC/ -3′IABkFQ. The amounts of HCV RNA were determined using the ddCt method with GAPDH as an internal control. For gene expression analysis, cDNAs were synthesized from the total RNA using a High Capacity RNA-to-cDNA kit (Applied Biosystems) according to the manufacturer's instructions. The specific primers used in this study are summarized in Supplementary Table 2. Mouse actin or human GAPDH mRNA was used as an internal control.

### Immunofluorescence staining

Cells fixed with 4% paraformaldehyde in PBS were permeabilized by incubating with 0.2% Triton X-100 in PBS for 15 min. After washing with PBS three times, the cells were incubated with anti-NS5A mouse monoclonal antibody (5A27, 1:2,000 dilution) or anti-FLAG mouse monoclonal antibody (M2, Sigma, 1:1,000 dilution) at room temperature for 1 h, washed three times with PBS, and incubated with Alexa Fluor 594-conjugated or Alexa Fluor 488-conjugated anti-mouse antibodies (1:2,000 dilution) at room temperature for 1 h. The nuclei were visualized with DAPI (Life Technologies), and the cells were observed under a fluorescence microscope (FV-1000, Olympus).

### Immunoprecipitation

Each of the cell lines were seeded on six-well plates (one plate for each cell line) and were infected with lentiviruses expressing HCV core. After 48 h, the cells were collected, and the cell lysates were incubated with anti-HA antibody (HA.11, Covance, 1 μl per sample) at 4 °C for 90 min and then further incubated with Protein G Sepharose 4B (GE Healthcare) at 4 °C for 90 min. The beads were washed three times with lysis buffer, boiled at 95 °C for 5 min, and subjected to western blotting.

### Western blotting

Cell lysates were prepared by incubation in Onyx lysis buffer consisting of 20 mM Tris-HCl (pH 7.4), 135 mM NaCl, 1% Triton X-100, 1% glycerol and protease inhibitor cocktail tablets (Roche Molecular Biochemicals) for 15 min at 4 °C. For the preparation of the liver tissues, frozen liver sections were homogenized in lysis buffer consisting of 20 mM Tris-HCl (pH 7.4), 135 mM NaCl, 1% NP-40, 1% glycerol and protease inhibitor cocktail tablets. These lysates were centrifuged at 15,000*g* for 5 min at 4 °C after sonication. The protein concentrations were determined using Bio-Rad Protein Assay Dye Reagent Concentrate (Bio-Rad). The supernatants were incubated with sample buffer at 95 °C for 5 min. For the detection of SPP and TRC8, the supernatants were mixed with sample buffer and incubated at 60 °C for 20 min to avoid aggregation. The samples were resolved by SDS–PAGE (Novex gels, Life Technologies) and were transferred onto nitrocellulose membranes (iBlot, Life Technologies). These membranes were blocked with PBS containing 5% skim milk and 1% Triton X-100 and then incubated with primary antibody (1:1,000 dilution) at 4 °C overnight. After washing, the membrane was incubated with horseradish peroxidase -conjugated secondary antibody (1:2,000 dilution) at room temperature for 2 h. The immune complexes were visualized with Super Signal West Femto substrate (Pierce) and were detected using an LAS-3000 image analyzer system (Fujifilm). Full size images of western blotting were shown in Supplementary Figs 8–10.

### Cell viability assay

Huh7 cells (2 × 10^4^ cells in 50 μl) were seeded on 96-well black plates (Corning), and serially diluted compound solution (50 μl) was added to each well. After 48 or 96 h, the cell numbers were determined using a CellTiter-Glo Luminescent Cell Viability Assay (Promega) according to the manufacturer's protocol.

### Dosing

Dosing of LY-411575 was performed as previously described^58^. Briefly, LY-411575 was formulated in 10 mg ml^−1^ solutions in 50% polyethylene glycol, 30% propylene glycol and 10% ethanol, and diluted in 0.4% methylcellulose for dosing. The mice were dosed orally once a day for 4 to 16 days with vehicle or 10 mg kg^−1^ LY-411575 (C57BL per 6 mice).

### ELISA

Blood was collected from mice under a fed condition. The insulin concentrations in the sera were determined by ELISA (Mercodia Ultrasensitive Mouse Insulin ELISA, Mercodia AB). The serum concentrations of β-amyloid 40 and 42 were quantified using a Human/Rat β-Amyloid (40) ELISA kit (WAKO) and Human/Rat β-Amyloid (42) ELISA kit (WAKO).

### Insulin-tolerance test

Freely fed mice were fasted during the test period, and human insulin (HumulinR, Eli Lilly) was intraperitoneally administered (1.5 U kg^−1^). Blood was obtained from the tip of the tail, and the glucose levels were determined at the indicated times using Glucose Pilot (Aventir Biotech). The glucose concentration was normalized to the baseline glucose concentration at the time of insulin administration.

### HE staining and Oil red O staining

Formalin-fixed livers were impregnated with 30% sucrose and frozen with Tissue-Tek OCT compound (Sakura Finetek). Ten-micrometer-thick frozen sections were stained with HE. To visualize lipids, frozen sections were stained with Oil Red O. The frozen sections were washed with running tap water for 5 min, rinsed with 60% isopropanol for 1 min and then stained with freshly prepared Oil Red O solution for 15 min. The sections were rinsed with 60% isopropanol, followed by light staining of the nuclei with hematoxylin (HE) for 15 s. The sections were observed under a microscope (Olympus).

### Lipid concentration in the liver

Frozen liver tissues were homogenized and extracted with chloroform-methanol (2/1, v/v) solution. The organic phase was dried and resolubilized in 2-propanol. The Tcho and TG levels were measured using commercially available kits (Sekisui Medical). FC and PL levels were also determined using commercially available kits (TOYOBO).

### Diet-induced liver steatosis

Twenty-four-week-old male mice were fed a CDAA diet (A0608301, ResearchDIETS Inc.) for 4 weeks. Accumulation of lipids in the liver was visualized by staining with Oil Red O solution.

## Additional information

**How to cite this article:** Aizawa, S. *et al*. TRC8-dependent degradation of hepatitis C virus immature core protein regulates viral propagation and pathogenesis *Nat. Commun.* 7:11379 doi: 10.1038/ncomms11379 (2016).

## Supplementary Material

Supplementary Information Supplementary Figures 1-11, Supplementary Tables 1-4 and Supplementary References.
