# Supplementary material for: TRC8-dependent degradation of hepatitis C virus immature core protein regulates viral propagation and pathogenesis
Source: Nat Commun. 2016 May 4;7:11379. doi: 10.1038/ncomms11379 (PMC4857398; doi:10.1038/ncomms11379)
Supplement: Supplementary Information — Supplementary Figures 1-11, Supplementary Tables 1-4 and Supplementary References. [file ncomms11379-s1.pdf]

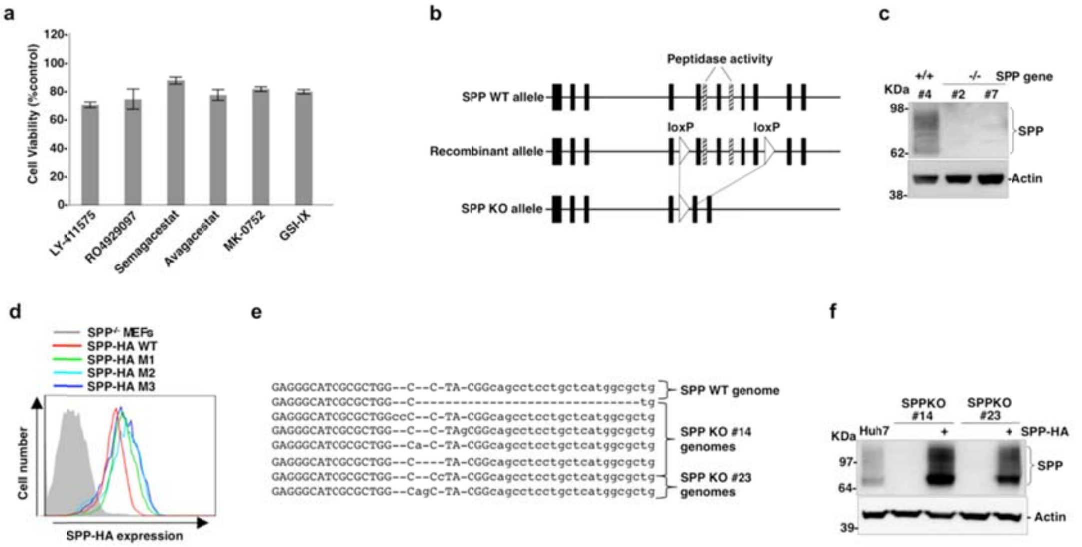

Supplementary Figure 1. (a) No compounds exhibited toxicity. Huh7 cells were incubated with 10 μM of the indicated compounds for 2 days, and the cell viability was determined using the CellTiter-Glo Luminescent Cell Viability Assay (Promega). The viability was normalized to that of the non-treated control. The data represent the mean ± SD of 2 independent experiments. **(b)** Schematic structures of the mouse SPP WT and loxP-targeted SPP loci. The black boxes indicate the exon sites, and the striped boxes indicate the peptidase activity sites of SPP. $SPP^{fl/+}$ mice were crossed with pGK-Cre mice to generate $SPP^{+/-}$ mice. (**c**) Confirmation of the lack of SPP protein in $SPP^{-/-}$ MEFs. The lack of SPP expression in MEFs established from $SPP^{-/-}$ embryos at E13.5 was confirmed by immunoblot analysis. (**d**) FACS analysis of the relevant protein expression of SPP and SPP mutants. Intracellular FACS analysis using anti-HA antibody revealed that SPP-HA expression in $SPP^{-/-}$ MEFs was comparable. (**e**) SPPKO Huh7 cells were generated using a CRISPR/Cas9 system. Mutations of the SPP gene in SPPKO Huh7 cells (#14 and #23) are shown. (**f**) The loss of the SPP protein in SPPKO Huh7 cell lines was confirmed by Western blotting. The data are representative of two independent experiments (c, d and f).

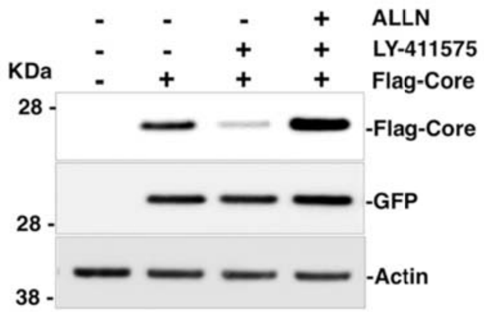

Supplementary Figure 2. The effects of LY-411575 and a proteasomal inhibitor on HCV core protein expression. Huh7 cells were infected with lentivirus expressing FLAG-core and then treated with LY-411575 (1.0 μM) for 10 h in the presence or absence of a proteasome inhibitor (ALLN, 5 μM). The cells were harvested and subjected to Western blotting using antibodies against the indicated proteins. The data are representative of three independent experiments.

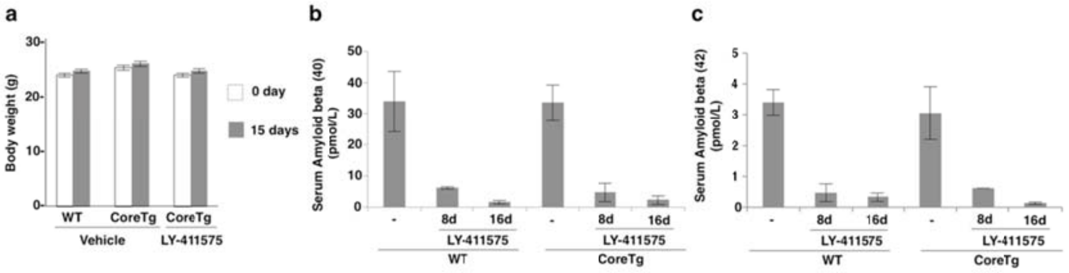

Supplementary Figure 3. (**a**) No weight loss occurred in mice following treatment with SPP. Mice (24-32 weeks old) were weighed before and after LY-411575 administration (n=6). (b, c) SPP inhibitor reduced the products of γ-secretase. Serum Aβ (40) (**b**) and Aβ (42) (**c**) levels were determined by ELISA (n=4). The data are shown as the mean ± SE from two independent measurements of at least three mice per genotype and time point.

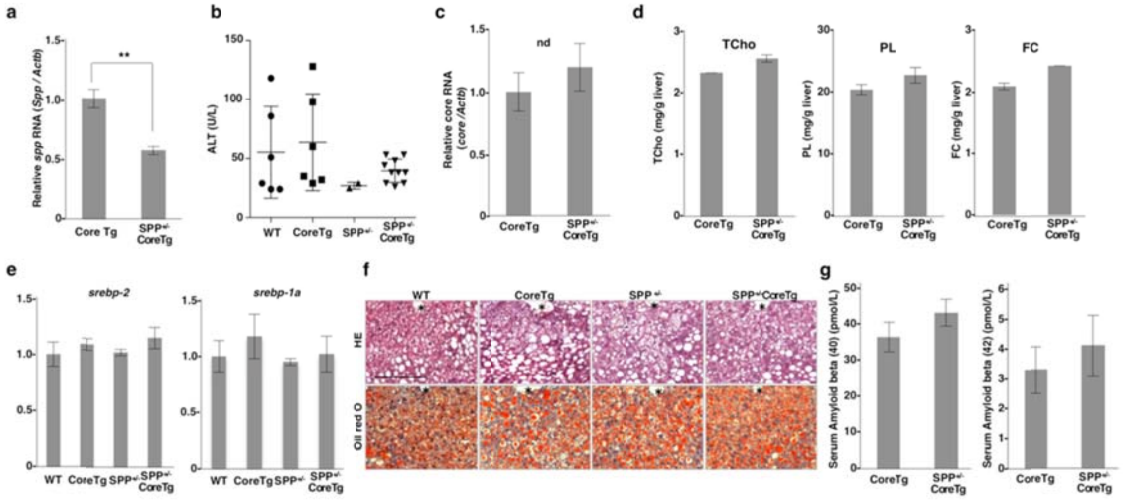

Supplementary Figure 4. (**a**) SPP mRNA expression in the livers of SPP$^{+/-}$CoreTg mice was lower than that of CoreTg mice. SPP mRNA expression in the livers of CoreTg and SPP$^{+/-}$CoreTg mice at the age of 24-26 weeks was determined by qPCR. (**b**) Haploinsufficiency of SPP did not result in a significant difference in the ALT levels. The ALT levels in the sera of the WT, CoreTg, SPP$^{+/-}$ and SPP$^{+/-}$CoreTg mice (n=3-8 in each genotype) were determined. (**c**) No significant difference was observed in core mRNA expression between CoreTg and SPP+/-CoreTg mice. Total RNA was obtained from the livers of CoreTg and SPP$^{+/-}$CoreTg male mice (n=4), and core mRNA expression was determined by qPCR. The data are shown as the relative expression levels after standardization by actin expression. (**d**) No differences were detected in the amounts of Tcho, PL and FC in the livers of CoreTg and SPP$^{+/-}$CoreTg mice. The amounts of Tcho, PL and FC in the livers of CoreTg and SPP$^{+/-}$CoreTg mice (24-26-weeks old, n=3 for each genotype) were determined. (**e**) SREBP-2 and SREBP-1a expression levels were unchanged in SPP$^{+/-}$CoreTg mice. Total RNA was prepared from the livers of WT, CoreTg, SPP$^{+/-}$ and SPP$^{+/-}$CoreTg mice (24-32-weeks old, male, n=4-6 for each genotype), and SREBP-2 and SREBP-1a expression levels were determined by qPCR. (**f**) SPP was not involved in diet-induced liver steatosis. WT, CoreTg, SPP$^{+/-}$ and SPP$^{+/-}$CoreTg mice (24-weeks old) were fed a CDAA diet for 4 weeks, and liver sections were then stained with HE (upper) and Oil Red O (lower). Scale bar: 100 μm. *: Central vein. (**g**) Haploinsufficiency of SPP was not involved in the production of Aβ (40) and Aβ (42). The serum Aβ (40) and Aβ (42) levels of CoreTg and SPP$^{+/-}$CoreTg mice at the age of 24-26 weeks were determined by ELISA. The data represent the mean ± SE from two independent measurements of at least three mice per genotype. Images are representative of four independent mice per genotype.

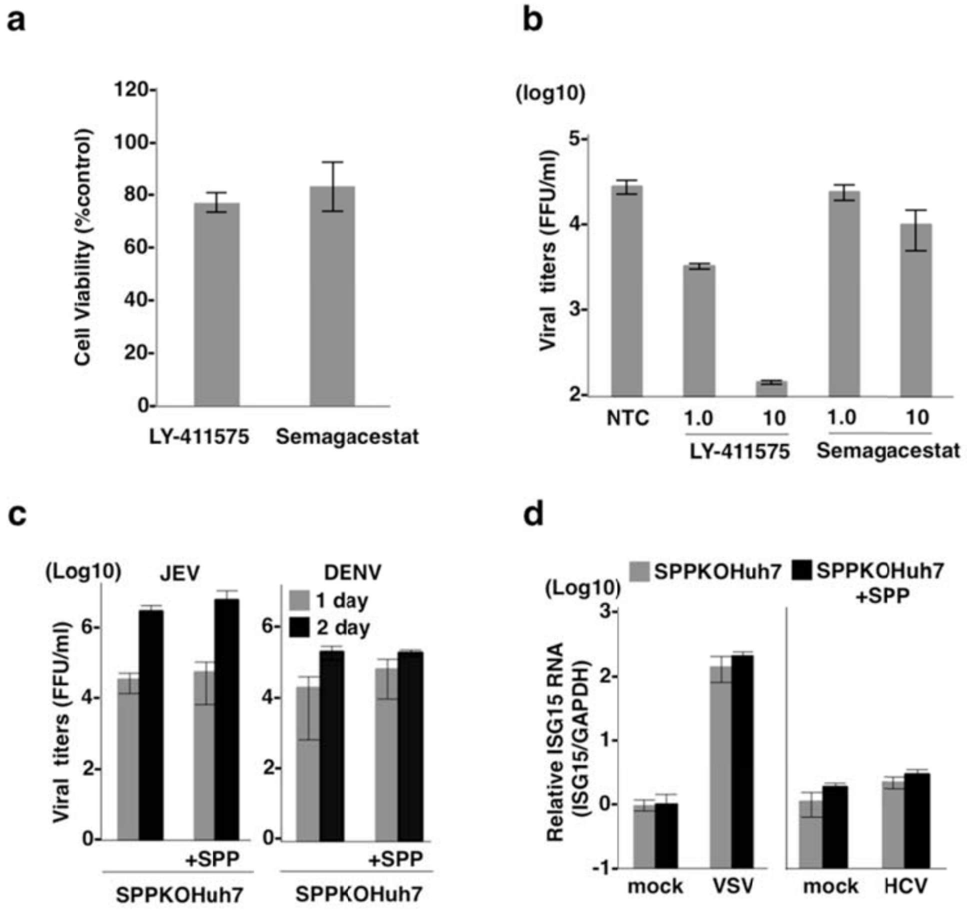

Supplementary Figure 5 (**a**) LY-411575 and semagacestat exhibited no toxicity. Huh7 cells were incubated with 40 μM of the indicated compounds for 4 days, and cell viability was determined using a CellTiter-Glo Luminescent Cell Viability Assay (Promega). The viability values were normalized to those of the non-treated control. The data represent the mean ± SD of 2 independent experiments. **(b)** LY-411575, but not semagacestat, suppressed HCV release. Huh7 cells infected with HCV at an moi of 0.5 were treated with LY-411575 or semagacestat (1 or 10 μM) for 4 days. Infectious titers in the culture supernatants were determined using a focus-forming assay. The data represent the mean ± SD of 2 independent experiments. **(c)** SPP expression had no effect on the production of DENV type 4 or JEV. SPPKOHuh7 cells and those with restored SPP expression were infected with JEV and DENV at an moi of 3, and infectious titers in the culture supernatants were determined using a focus-forming assay at 4 days post-infection. The data represent the mean ± SD of 2 independent experiments. (**d**) SPP was not involved in ISG15 expression. SPPKOHuh7 cells and those with restored SPP expression were infected with VSV (left) and HCV (right), and ISG15 expression levels were then determined by qPCR at 6 and 24 h post-infection, respectively. The data represent the mean ± SD of 2 experiments performed with a cell line representative of SPPKOHuh7 cells.

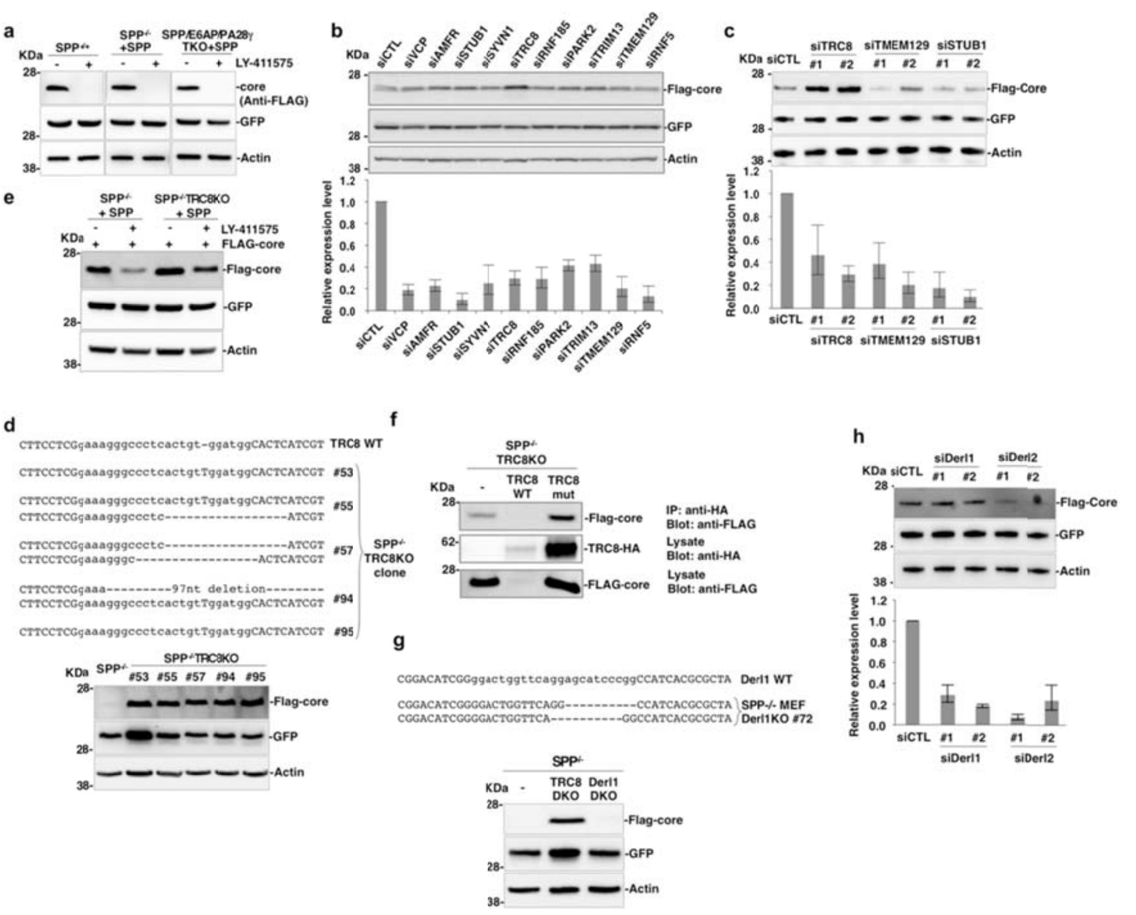

Supplementary Figure 6 (**a**) Neither E6AP nor PA28γ were involved in the core degradation induced by an SPP inhibitor. SPP$^{-/-}$ and SPP/PA28γ/E6APTKO MEFs with restored SPP expression were infected with lentivirus expressing FLAG-core, treated with LY-411575 (1.0 μM) at 48 h post-infection and subjected to immunoblotting. (**b**) TRC8 knockdown impaired core degradation in SPPKOHuh7 cells. SPPKOHuh7 cells were transfected with FLAG-core 24 h after transfection of siRNAs targeting the ERAD-associated E3 ubiquitin ligases AMFR, STUB1, SYVN1, TRC8, RNF185, PARK2, TRIM13, TMEM129 and RNF5, and VCP (AAA-ATPase p97/VCP) and were subjected to immunoblotting using antibodies against the indicated proteins (upper). Amounts of total RNA extracted from SPPKOHuh7 cells transfected with siRNA and the knockdown of each targeting gene were verified by qPCR (lower). (**c**) TRC8 knockdown impaired core degradation in SPPKO Huh7 cells. SPPKOHuh7 cells transfected with FLAG-core at 24 h post-transfection of two different siRNAs targeting TRC8, TMEM129 or STUB1 were subjected to immunoblotting using antibodies against the indicated proteins (upper), and knockdown of each targeting gene was verified by qPCR (lower). (**d**) Establishment of SPP$^{-/-}$TRC8KO MEFs. Mutations of the TRC8 gene in SPP$^{-/-}$ MEFs (#53, #55, #57, #94 and #95) are shown (top). SPP$^{-/-}$ MEFs or 5 different SPP$^{-/-}$TRC8KO MEF clones were infected with lentivirus expressing FLAG-core and were then subjected to immunoblotting using antibodies against the indicated proteins. (**e**) TRC8 was involved in core degradation induced by an SPP inhibitor. SPP$^{-/-}$ and SPP$^{-/-}$TRC8KO MEFs with restored SPP expression expressing FLAG-core were treated with LY-411575 (1.0 μM) at 48 h post-infection and were then subjected to immunoblotting. (**f**) A TRC8 mutant interacted with HCV immature core protein. SPP$^{-/-}$TRC8KO MEFs and those restored with wild-type or mutant TRC8 were infected with lentivirus expressing FLAG-core, and the cell lysates were immunoprecipitated with anti-HA antibody. The immunoprecipitated samples were

subjected to SDS-PAGE and Western blotting. (**g**) Generation of SPP$^{-/-}$Derl1KO MEFs. Mutations of the Derl1 gene in SPP$^{-/-}$ MEFs (#72) are shown (top). SPP$^{-/-}$Derl1KO MEFs and SPP$^{-/-}$ MEFs were infected with a lentivirus expressing FLAG-core and were then subjected to immunoblotting. (**h**) Neither Derl1 nor Derl2 participates in immature core protein degradation. SPPKOHuh7 cells were transfected with FLAG-core at 24 h post-transfection of siRNAs targeting Derl1 and Derl2, which included two independent targeting sequences, and were then subjected to immunoblotting using antibodies against the indicated proteins (upper). Amounts of total RNA extracted from SPPKOHuh7 cells transfected with siRNA and the knockdown of each targeting gene were verified by qPCR (lower). The data are representative of two (a and c-h) and three (b) independent experiments. qPCR data represent the mean ± SD of 2 independent experiments (b, c and h).

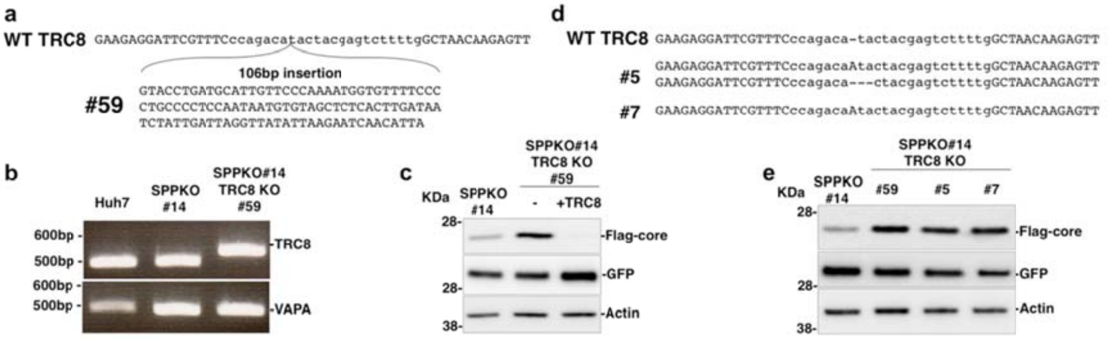

Supplementary Figure 7 (**a**) Mutations of the TRC8 gene in SPPKO Huh7 cells (#59) are shown. (**b**) Confirmation of TRC8 deficiency by PCR. Genomic DNA was amplified using specific primers for TRC8 and VAPA. The PCR product of the TRC8 gene in SPP/TRC8DKO Huh7 cells was approximately 100 bp longer than that in the parental Huh7 cells (lower). The primers for VAPA were 5'-GTTGAGTCAGTTGTGGGACC-3' and 5'-GCAAGAAAGCAGCCGGGAAA-3'. (**c**) TRC8 was involved in immature core degradation in SPPKOHuh7 cells. SPPKO Huh7, SPP/TRC8DKO Huh7 and SPP/TRC8DKO Huh7 cells restored by HA-tagged TRC8 expression were infected with a lentivirus expressing FLAG-core and were then subjected to immunoblotting. (**d**) Mutations of the TRC8 gene in SPPKO Huh7 cells (#5, #7) are shown. (**e**) TRC8 was involved in immature core degradation in SPPKOHuh7 cells. SPPKO Huh7 and SPP/TRC8DKO Huh7 (#59, #5 and #7) were infected with a lentivirus expressing FLAG-core and were then subjected to immunoblotting. The data are representative of two independent experiments.

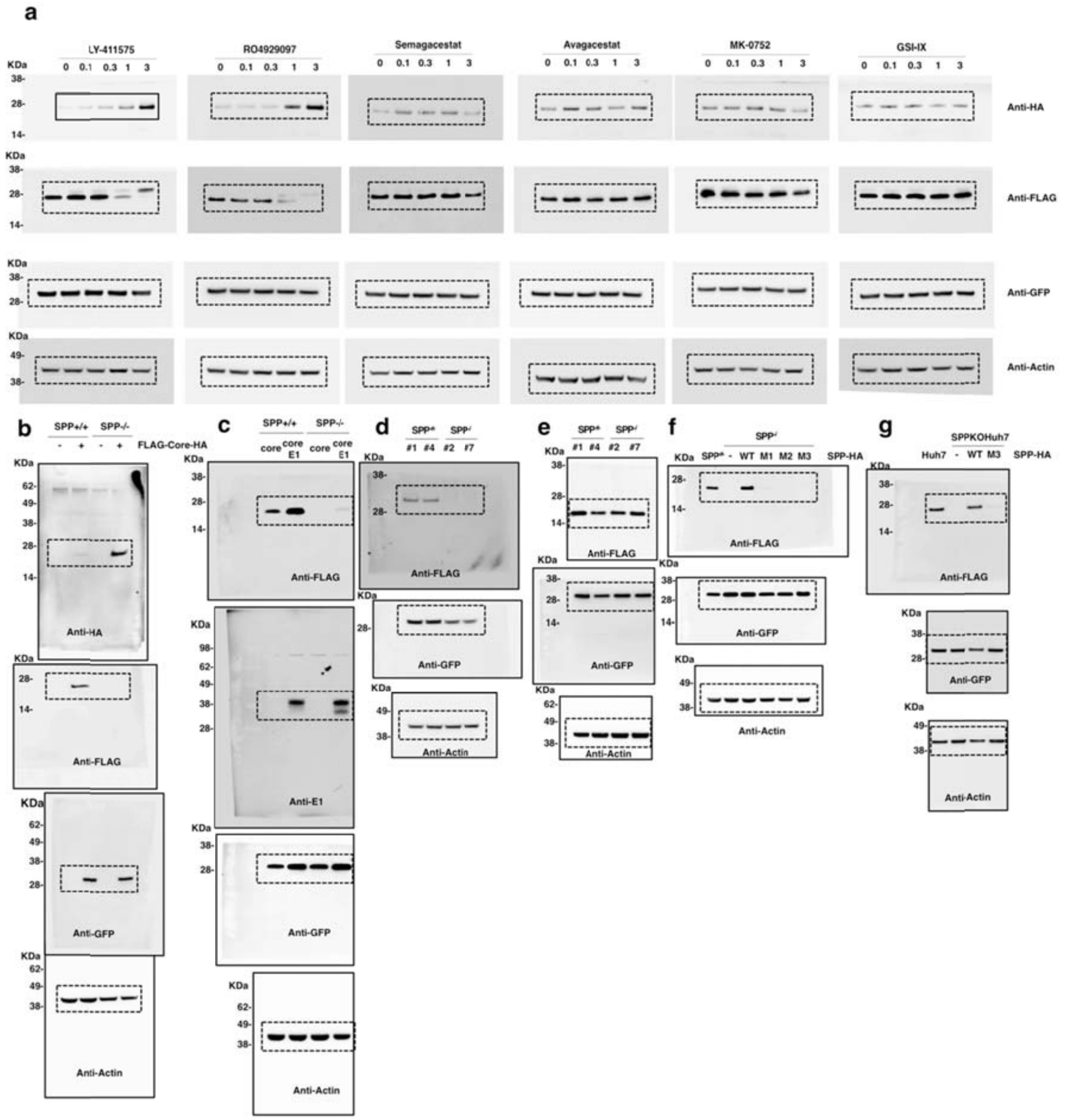

Supplementary Figure 8 : Scanned images of Western blotting data used in Figure 1. (a) Fig. 1b (b) Fig. 1e (c) Fig. 1f (d) Fig. 1g (e) Fig. 1h (f) Fig. 1i (g) Fig. 1j

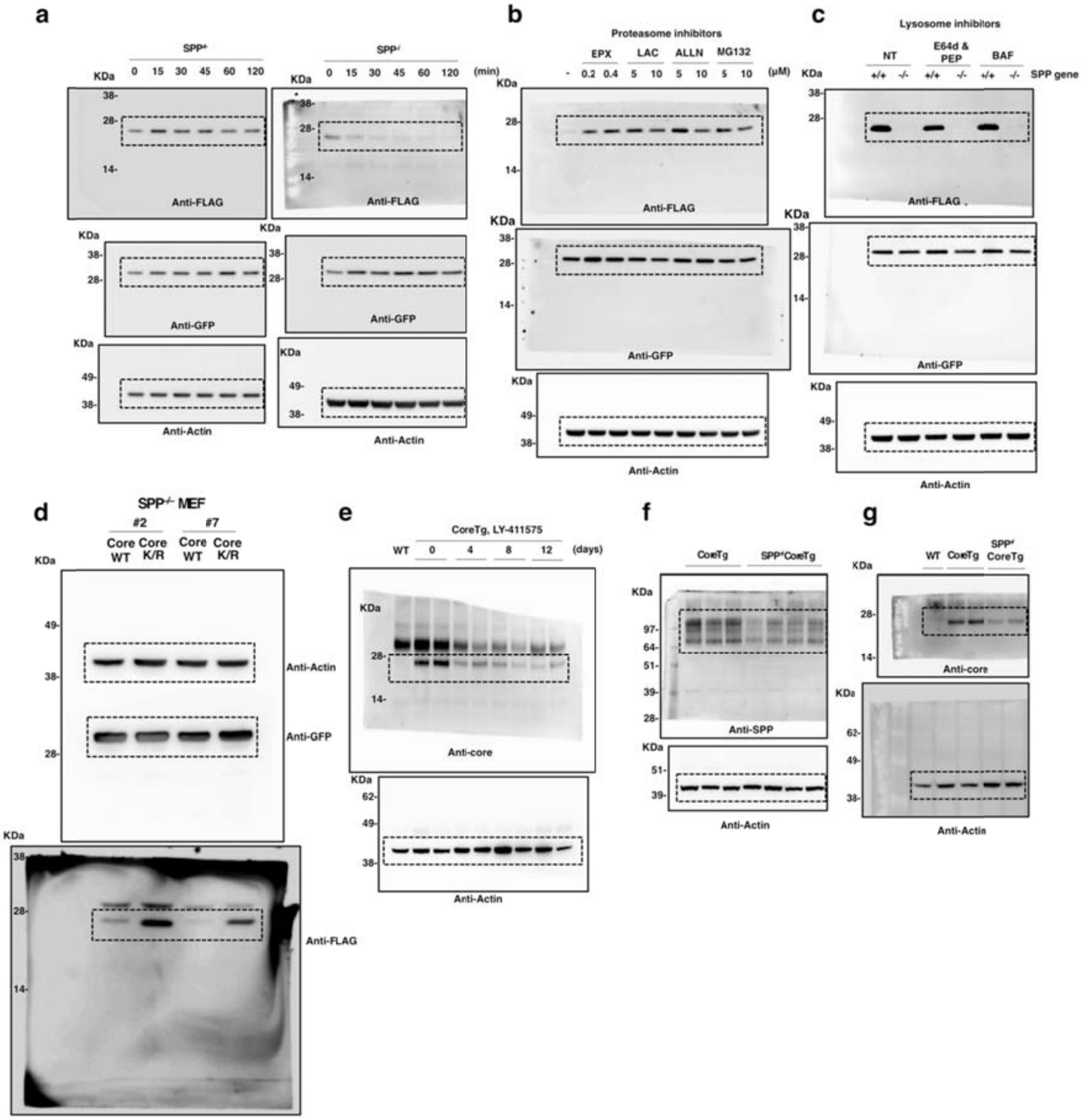

Supplementary Figure 9 : Scanned images of Western blotting data used in Figure 2-4. (a) Fig. 2a (b) Fig. 2b (c) Fig. 2c (d) Fig. 2d (e) Fig. 3c (f) Fig. 4a (g) Fig. 4b

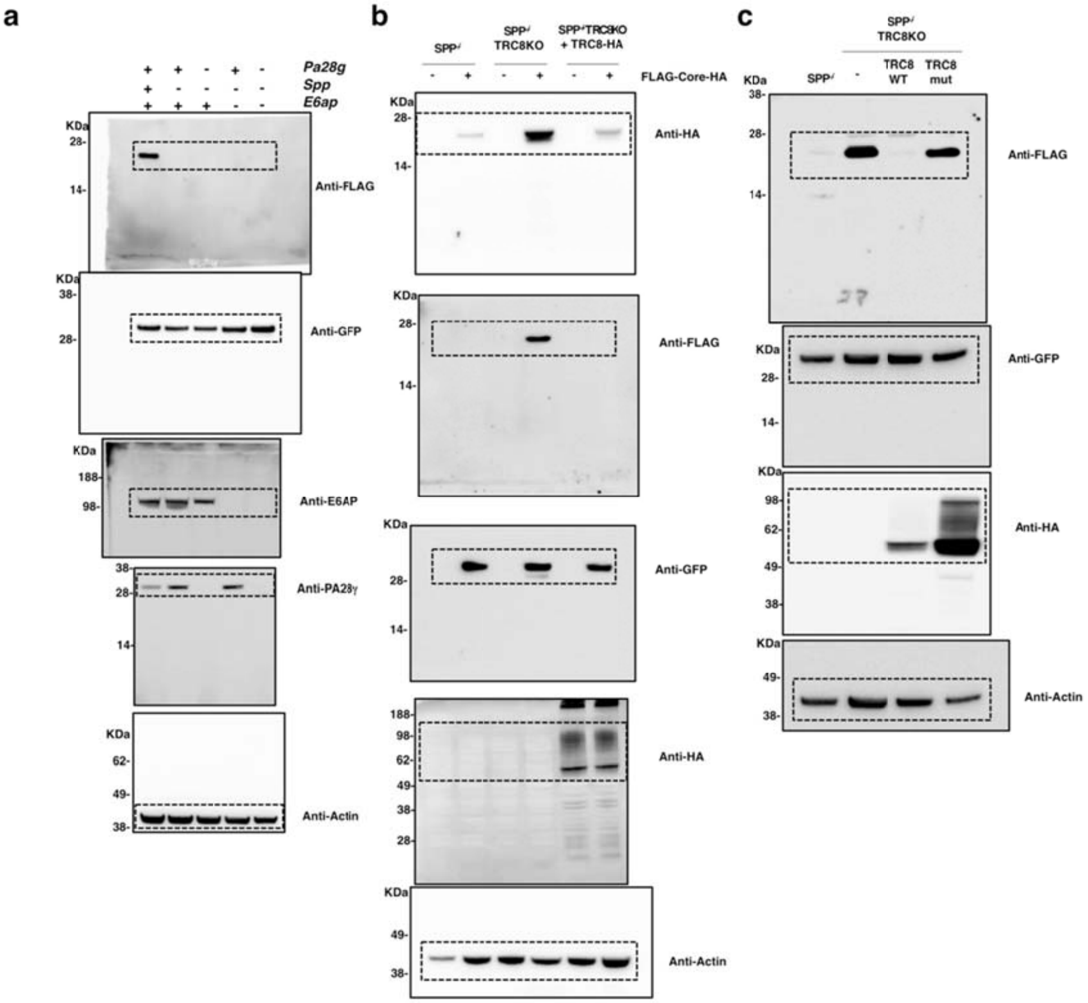

Supplementary Figure 10 : Scanned images of Western blotting data used in Figure 6. (a) Fig. 6b (b) Fig. 6c (c) Fig. 6d

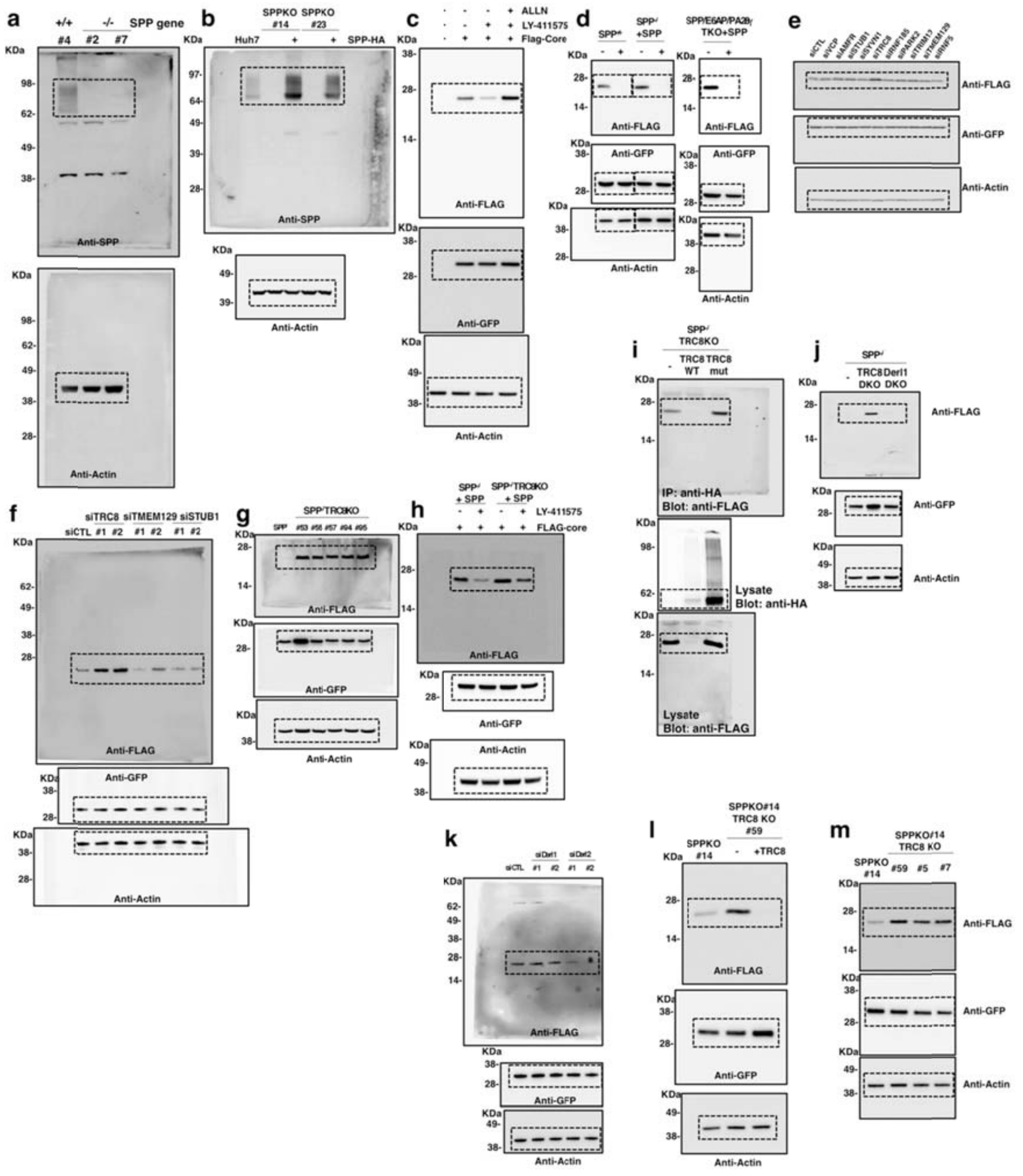

Supplementary Figure 11 : Scanned images of Western blotting data used in Supplementary Figures. (a) Fig. S1c (b) Fig. S1f (c) Fig. S2 (d) Fig. S6a (e) Fig. S6b (f) Fig. S6c (g) Fig. S6d (h) Fig. S6e (i) Fig. S6f (j) Fig. S6g (k) Fig. S6h (l) Fig. S7c (m) Fig. S7e

Supplementary Table 1

Primers used in this study

Primer number	Primer name	Template	sequence	Resulting plasmids
#165	IRES-puro Fw	pIRES-puro3 (Clontech)	ttgttagacaGGATCCaatGAATTCACGCGTGCTAGCatagataact gatccagtgtg	FUIPW
#166	IRES-puro Rv		gcttgatatcGAATTatccctctacaaatgtggtatgg	FUIPW
#349	IRES-GFP Fw	pMIG[1]	ttgttagacaGGATCCaatGAATTCttagatctctcgaggttaacgaattcc	FUIGW
#347	IRES-GFP Rv		gcttgatatcGAATTttacttgtacagctcgtcca	FUIGW
#1089	SPP Fw	pcDNA3.1 SPP-HAer[2]	aGGATCCaatGAATTCaccATGGACTCGGCCCTCAGCG	FUIPW SPP-HAer
#1090	HAer Rv		TAGCACGCGTgaattcCTATTTCTCTTTCTTGATGG	FUIPW SPP-HAer
#1439	FLAG Fw		ttgttagacaGGATCCaccATGGACTACAAAGACGATGA	FUIGW FLAG-EHcV core<br>FUIGW FLAG-JEV core<br>FUIGW FLAG-HCV core<br>FUIGW FLAG-core-HA<br>FUIGW FLAG-core-E1
#951	EHcV core Rv	pcDNA3.1 EHcV core[3]	aGAATTCattGGATCCCTAGACCTCACCAAAAGATACGGC	FUIGW FLAG-EHcV core
#1381	JEV core Rv	pCAG/FLAG-JEC [4]	aGAATTCattGGATCCCTAtCTTTTGTTTTGCTTTCTGCC	FUIGW FLAG-JEV core
#1516	HCV core Rv	pcDNA3.1 FLAG-core-HA[2]	aGAATTCattGGATCCttaagcggaagctgggatgg	FUIGW FLAG-HCV core
#1440	HA Rv	pcDNA3.1 FLAG-core-HA[2]	cGAATTCattGGATCCTTAGATGGCGTAGTCTGGGA	FUIGW FLAG-core-HA
#1578	E1 Rv	pcDNA3.1 FLAG-core-E1-HA[2]	aGAATTCattGGATCCttaCCCGTCAACGCCGGCAA	FUIGW FLAG-core-E1
#1621	TRC8 Fw	cDNA of Huh7	ttgttagacaGGATCCaccatggcggccgtggggccccc	FUIPW TRC8-HA<br>FUIPW TRC8mut-HA
#1624	TRC8-HA Rv		TGAATTCattggatccttaGATGGCGTAGTCTGGGACGTCGT ATGGGTATCgaattctgtcagtatcatcattaaattcttca	FUIPW TRC8-HA<br>FUIPW TRC8mut-HA
#1622	TRC8mut Rv		ataggcgattgcaGCtacatcatttatttcttgta	FUIPW TRC8mut-HA
#1623	TRC8mut Fw		tgtaGCtgcaatcgcctatcatgagtttacaacat	FUIPW TRC8mut-HA
#1106	Core Fw OSF	pcDNA3.1 FLAG-core-HA[2]	CGATGACAAGgaattcagcacaaatcctaaacccca	pCAGGS OSF-core
#1107	Core Rv		CGATGAGCTCGAATTCttaagcggaagctgggatgg	pCAGGS OSF-core
#1110	OSF Fw	pCAG OSF[5]	attttggcaaagaattaccATGGCTAGCTGGAGCCACCC	pCAGGS OSF-core
#1111	OSF Rv core		tttaggatttgtgctgaattcCTTGTCATCGTCATCCTTGT	pCAGGS OSF-core

Supplementary Table 2

DNA oligos for generating gene-knockout cell lines.

	Target sequence for Cas9<br>Forward primer for cloning into pCAG EGxxFP and sequencing<br>Reverse primer for cloning into pCAG EGxxFP and sequencing
Human SPP	5'-GCCCTCAGCGATCCGCATAACGG-3'<br>5'-CAACCACTGAGGATCCCACGTCACTTCCTGTTGCCTTAG-3'<br>5'-TGCCGATATCGAATTCGGTTGGATGGTAGTTAAGAGAGGA-3'
Mouse PA28γ	5'-GACACGGCCCGGCCTGGCCATGG-3'<br>5'-CAACCACTGAGGATCCCCAGGAACCTGTAGAACTGAG-3'<br>5'-TGCCGATATCGAATTCCTATACGGTCCTCTCCAATGC-3'
Mouse E6AP	5'-CCTCATCCCTCCAAGAAAGGAGC-3'<br>5'-CAACCACTGAGGATCCTTGCCTTATCTGTTGGGCTTTG-3'<br>5'-TGCCGATATCGAATTCCCAGTCCTTTACCACAGTTTACCAG-3'
Mouse Derl1	5'-GGACTGGTTCAGGAGCATCCCGG-3'<br>5'-CAACCACTGAGGATCCTGATCGCTGCCTAGCTTGTC-3'<br>5'-TGCCGATATCGAATTCCACTACAGATCCCACCAGCC-3'
Mouse TRC8	5'-GAAAGGGCCCTCACTGTGGATGG-3'<br>5'-CAACCACTGAGGATCCGCCTTTCTATTAGCTGCAACTTC-3'<br>5'-TGCCGATATCGAATTCCTGCCAAGACAAGCACTGTG-3'
Human TRC8	5'-CCAGACATACTACGAGTCTTTTG-3'<br>5'-CAACCACTGAGGATCCGTCCTGGCAGTGAAACTGAAG-3'<br>5'-TGCCGATATCGAATTCCAACAAAGCCAAGACGCCTG-3'

Supplementary Table 3

siRNAs used in this study

	siRNA ID	Sequence (+ TT)
siCTL		Silencer Select Negative Control No. 1 siRNA (cat no. 4390843)
siVCP	s14765	**GAAUAGAGUUGUUCGGAAUTT**
siAMFR	s1322	**GCUCUGCAAGGAUCGAUUUTT**
siSTUB1-1	s195025	**GUCUGUUCGUGGGCCGAAATT**
siSTUB1-2	s195027	**CCAAUCUGCAGCGAGCUUATT**
siSYVN1	s39021	**GCAUUGUCUCUCUUAUGUUTT**
siTRC8-1	s22178	**GGGAAAAGCUUGACGAUUATT**
siTRC8-2	s22179	**GUAUCGAAUUUACGGAUUATT**
siRNF185	s40665	**CAUCAGUGGUUGGAGACCATT**
siPARK2	s224170	**CCAACUCCUUGAUUAAAGATT**
siTRIM13	s19898	**GAGUUUAGAGAGAAAAUCATT**
siTMEM129-1	s40916	**GACGUGCACCUGACUGUGATT**
siTMEM129-2	s40917	**CCCGUGUGAUUGUGACAGATT**
siRNF5	s12076	**CCACCGUCUUCAAUGCCCATT**

Supplementary Table 4

Primers for qPCR

	Forward primer<br>Reverse primer
Human GAPDH	5’-TGTAGTTGAGGTCAATGAAGGG-3’<br>5’- ACATCGCTCAGACACCATG-3’
Human ISG15	5’- AGCGAACTCATCTTTGCCAGTACA-3’<br>5’- CAGCTCTGACACCGACATGGA-3’
Mouse β-actin	5′ - TTGCTGACAGGATGCAGAAG -3′<br>5′ - GTACTTGCGCTCAGGAGGAG -3′
Mouse SPP	5′ - TTTCTTCGTGCTGGGGATCC -3′<br>5′ - CCTGTGTGAAGAGCAGCTGA -3′
Mouse SREBP-1a	5′ - CACAGCGGTTTTGAACGAC -3′<br>5′ - CTGGCTCCTCTTTGATCCCA -3′
Mouse SREBP-1c	5′ - ACGGAGCCATGGATTGCACATTTG -3′<br>5′ - TACATCTTTAAAGCAGCGGGTGCCGATGGT -3′
Mouse SREBP-2	5′ - ACCATTCTCCAGCAGTTCCGT -3′<br>5′ -CCTCTCACAGTGACAGAAGGAGTT -3′
HCV core	5′ - GTCAGATCGTCGGTGGAGTT -3′<br>5′ - GAGCCTTGGGGATAGGTTGT -3′
Human VCP	5′ - ATTGATCCTGCCATCCTCAG -3′<br>5′ - AAGTCCACATCCTTGGCAAC -3′
Human AMFR	5′ - CAGCTGCGTTACCTGTTTCA -3′<br>5′ - TCATTGTTGACAGCCAGCTC -3′
Human STUB1	5′ - TCATTGTTGACAGCCAGCTC -3′<br>5′ - GCTCCTCAATGCTGTTCCAG -3′
Human SYVN1	5′ - GTGATGGGCAAGGTGTTCTT -3′<br>5′ - CAAGTCTCTGTGACGGCGTA -3′
Human TRC8	5′ - GCATCGTGCTCCAGATCTTC -3′<br>5′ - TGCAGCTAACAGAAAGGCTGA -3′
Human RNF185	5′ - GTCGGAGGTCTTACCCAACA -3′<br>5′ - TCAGCGAAGCTCTGAGTGAA -3′
Human PARK2	5′ - GCATCTTCCAGCTCAAGGAG -3′<br>5′ - GCATCTTCCAGCTCAAGGAG -3′
Human TRIM13	5′ - ACCCTGCCAAATACATCAGC -3′<br>5′ - ACCCTGCCAAATACATCAGC -3′
Human TMEM129	5′ - TGACATCTGGCTGAACTCCA -3′<br>5′ - GGACACAGCGGTTTTGAACG -3′
Human RNF5	5′ - TGGGATCAGCAGAGAGAAGG -3′<br>5′ - GGTATCACCAAATGGCTGGA -3′
Human BIP	5’- CGAGGAGGAGGACAAGAAGG -3’<br>5’- CACCTTGAACGGCAAGAACT -3’
Human CHOP	5’- GCACCTCCCAGAGCCCTCACTCTCC -3’<br>5’- GTCTACTCCAAGCCTTCCCCCTGCG -3’
Human RNF5	5′ - TGGGATCAGCAGAGAGAAGG -3′<br>5′ - GGTATCACCAAATGGCTGGA -3′

Reference for Supplementary information

1. Chen, L. *et al.* Differential Targeting of Prosurvival Bcl-2 Proteins by Their BH3-Only Ligands Allows Complementary Apoptotic Function. *Mol. Cell* **17,** 393–403 (2005).
2. Okamoto, K., Moriishi, K., Miyamura, T. & Matsuura, Y. Intramembrane proteolysis and endoplasmic reticulum retention of hepatitis C virus core protein. *J. Virol.* **78,** 6370–6380 (2004).
3. Tanaka, T. *et al.* Hallmarks of Hepatitis C Virus in Equine Hepacivirus. *J. Virol* **88,** 13352–13366 (2014).
4. Mori, Y. *et al.* Processing of Capsid Protein by Cathepsin L Plays a Crucial Role in Replication of Japanese Encephalitis Virus in Neural and Macrophage Cells. *J. Virol.* **81,** 8477–8487 (2007).
5. Morita, E. *et al.* Identification of human MVB12 proteins as ESCRT-I subunits that function in HIV budding. *Cell Host and Microbe* **2,** 41–53 (2007).
